# **PD-L1 siRNA hitched polyethyleneimine-elastase constituting nanovesicle induces tumor immunogenicity and PD-L1 silencing for synergistic antitumor immunotherapy**

**DOI:** 10.1186/s12951-024-02700-4

**Published:** 2024-07-27

**Authors:** Li Du, Yao Gong, Xiaoying Zhang, Jide Sun, Fengxia Gao, Meiying Shen, Huili Bai, Tiantian Yang, Xiaoxue Cheng, Siqiao Li, Jian Peng, Zhangling Liu, Shijia Ding, Junman Chen, Wei Cheng

**Affiliations:** 1 The Center for Clinical Molecular Medical Detection, The First Affiliated Hospital of Chongqing Medical University, Chongqing, 400016 China; 2 Biobank, The First Affiliated Hospital of Chongqing Medical University, Chongqing, 400016 China; 3 Department of Laboratory Medicine, The First Affiliated Hospital of Chongqing Medical University, Chongqing, 400016 China; 4 Department of Endocrine and Breast Surgery, The First Affiliated Hospital of Chongqing Medical University, Chongqing, 400016 China; 5 Department of Forensic Medicine, Faculty of Basic Medical Sciences, Chongqing Medical University, Chongqing, 400016 China; 6 Key Laboratory of Clinical Laboratory Diagnostics (Ministry of Education), College of Laboratory Medicine, Chongqing Medical University, Chongqing, 400016 China

**Keywords:** Elastase, Polyethyleneimine, PD-L1 siRNA, Liposomes, Anti-tumor immune response

## Abstract

**Background:**

PD-1/PD-L1 blockade has become a powerful method to treat malignant tumors. However, a large proportion of patients still do not benefit from this treatment, due to low tumor immunogenicity and low tumor penetration of the agents. Recently, neutrophil elastase has been shown to induce robust tumor immunogenicity, while the insufficient enzyme activity at the tumor site restricted its anti-tumor application. Here, we designed polyethyleneimine-modified neutrophil elastase (PEI-elastase) loaded with PD-L1small interfering RNA (PD-L1 siRNA) for improving enzymatic activity and delivering siRNA to tumor, which was expected to solve the above-mentioned problems.

**Results:**

We first demonstrated that PEI-elastase possessed high enzymatic activity, which was also identified as an excellent gene-delivery material. Then, we synthesized anti-tumor lipopolymer (P-E/S Lip) by encapsulating PEI-elastase and PD-L1siRNA with pH-responsive anionic liposomes. The P-E/S Lip could be rapidly cleaved in tumor acidic environment, leading to exposure of the PEI-elastase/PD-L1 siRNA. Consequently, PEI-elastase induced powerful tumor immunogenicity upon direct tumor killing with minimal toxicity to normal cells. In parallel, PEI-elastase delivered PD-L1siRNA into the tumor and reduced PD-L1 expression. Orthotopic tumor administration of P-E/S Lip not only attenuated primary tumor growth, but also produced systemic anti-tumor immune response to inhibit growth of distant tumors and metastasis. Moreover, intravenous administration of P-E/S Lip into mice bearing subcutaneous tumors leaded to an effective inhibition of established B16-F10 tumor and 4T1 tumor, with histological analyses indicating an absence of detectable toxicity.

**Conclusions:**

In our study, a protease-based nanoplatform was used to cooperatively provoke robust tumor immunogenicity and down-regulate PD-L1 expression, which exhibited great potential as a combination therapy for precisely treating solid tumors.

**Graphical Abstract:**

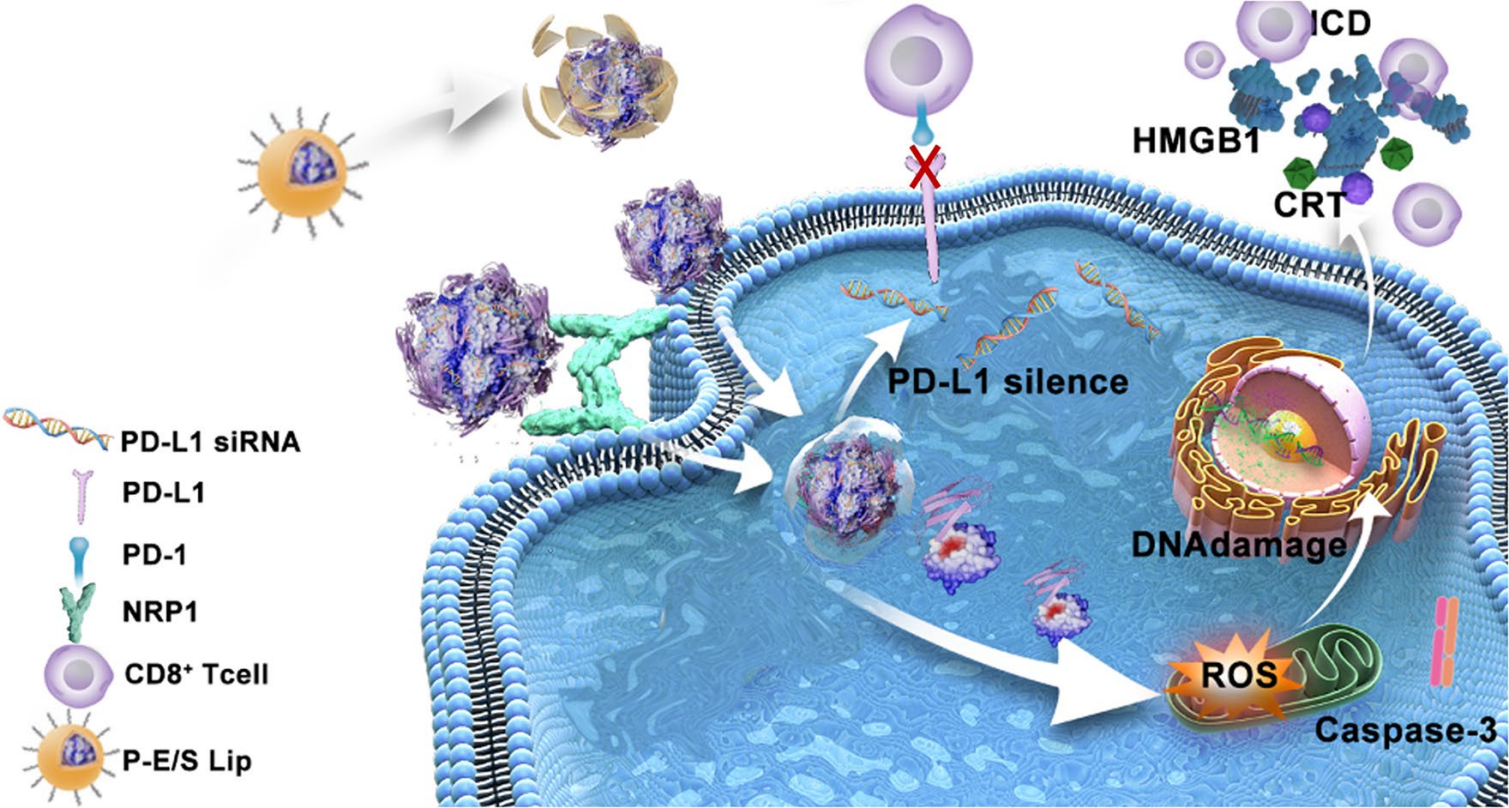

**Supplementary Information:**

The online version contains supplementary material available at 10.1186/s12951-024-02700-4.

## Introduction

Immune checkpoint blockade (ICB) has proven efficacious in multiple solid tumors. Among them, PD-1 immune checkpoint inhibitor has significantly elevated patient survival rates, thereby sparking widespread interest [1]. However, only a subset of patients benefit from anti-PD-1/PD-L1 treatment, and the overall response rate is relatively low [2]. A major mechanism of PD-1/PD-L1 immune resistance is low intrinsic immunogenicity, where tumor cells display limited level of immunogenic tumor antigens [3]. Another significant cause is that the use of PD-1/PD-L1 antibodies often suffer from low tumor penetration, and immune-related adverse effects. Therefore, it’s urgent to develop new immunotherapeutic approaches or combinatorial regimens to increase therapeutic efficacy.

Currently, some therapies have been shown to induce certain tumor immunogenicity. However, those therapies including conventional radiotherapy and chemotherapy always damage the host immune cells and impair the anti-tumor immunity. Neutrophil elastase (NE) is a serine protease stored in the azurophil granules of neutrophils [4]. Previous studies have confirmed that NE could increase major histocompatibility complex class 1 molecule (MHC-1) expression and tumor-associated antigen (TAA) presentation, leading to highly immunogenic tumors [5–7]. Recent studies have further proven that NE could mediate tumor killing directly while sparing non-cancer cells, which in turn enhanced tumor immunogenicity and facilitated tumor infiltration of CD8^+^ T cells to attack tumors within functional immune systems [8, 9]. These studies indicate that NE is a potential agent suitable for inducing tumor immunogenicity without immune cell damage. However, NE shows reduced tumor-killing activity at the tumor site, because serum proteinase inhibitors lead to decreased enzyme activity and stability. In addition, NE is an alkaline serine protease which optimal activity is in the alkaline pH range [10, 11]. The acidic tumor microenvironment (pH 6.5 to 6.8) inhibits the enzymatic activity and impedes anti-tumor activity.

Another important issue is that PD-1/PD-L1 antibodies have difficulty penetrating tumor tissues because of their enormous size. In addition, the antibodies only block the PD-L1 on cell membranes, which could be compensated by the constant expression of proteins in the cytoplasm [12–14]. To overcome these issues, RNA interference–based PD-1/PD-L1 knockdown provides an alternative approach to enhance T cell–mediated tumor killing [15, 16]. Compared to PD-L1 antibody, PD-L1 small interfering RNA (PD-L1 siRNA) can penetrate the tissue efficiently and down-regulate the PD-L1 protein from the intracellular source [17]. However, lack of suitable PD-L1 siRNA delivery systems has been limiting development of anti-tumor applications.

Polyethylenimine (PEI) is a well-known cationic polymer with buffering capacity [18]. Explorations have documented protease modification with positively charged PEI can shift the optimum pH of the proteases toward the acidic range. For example, immobilization of urease onto egg shell membrane through PEI cross linking shifted the optimum pH of enzyme from 8.0 to 7.5 [19]. We hypothesized that covalent modification of NE with PEI could shift the optimum pH of NE toward the acidic direction, thus improving elastase’s catalytic activity and stability under acidified conditions. Meanwhile, PEI is also a well-known cationic carrier for the delivery of nucleic acids [20, 21]. PEI crosslinking or linking to targeting ligands has been shown as an efficient and safe delivery material [22–24]. For example, PEI modified with phenylboronic acid (PBA) could be effectively utilized for the delivery of therapeutic nucleic acids [25]. Fluorocarbon chains grafted on PEI could efficiently mediate the transmembrane delivery of protein into the cell [21, 26]. NE was shown to target tumor cells via neuropilin-1 (NRP1), which is highly expressed on surface of many tumor cells [5–7]. We speculated that NE-grafted PEI could be used as a tumor-targeting delivery vehicle to deliver PD-L1siRNA.

**Scheme 1:**
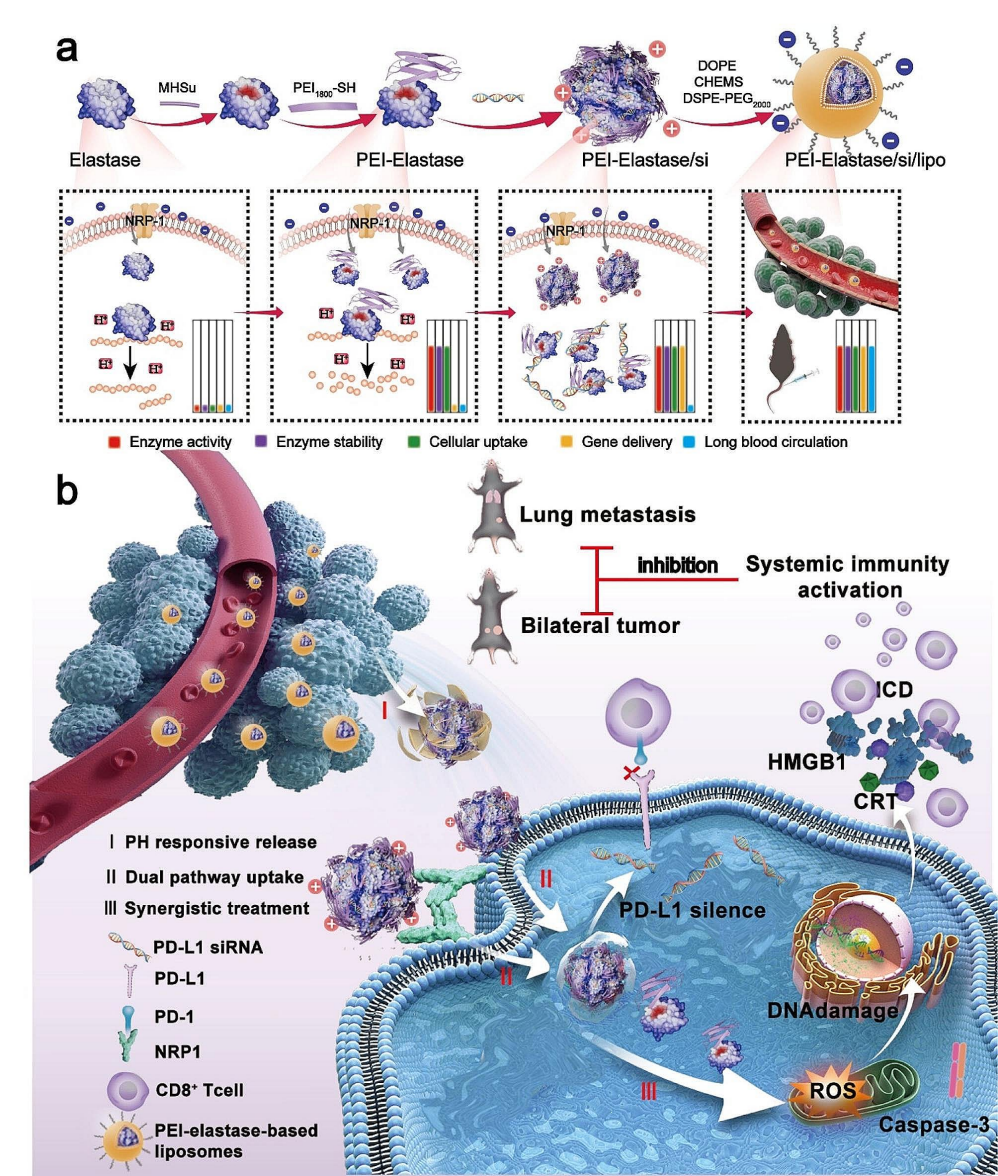
Diagram of the design and synthesis of PEI-elastase-based liposomes and their function. (**a**) Schematic diagram of PEI-elastase and P-E/S Lip synthetic processes. (**b**) Schematic illustration of anti-tumor characteristics of P-E/S Lip

Herein, PEI modified elastase/PD-L1siRNA nanomaterials with liposome encapsulation (named as P-E/S Lip) were constructed for enhancing tumor immunogenicity and downregulating PD-L1 expression. PEI-elastase was first synthesized via a michael addition reaction and an amide coupling reaction. Since PEI-elastase is positively charged under physiological conditions, it can load with PD-L1siRNA to form complex micelles by electrostatic interactions. Then, a layer of pH-responsive liposomes was coated onto the micelles with a negatively charged surface (Scheme.1a). The anionic liposomes offered the advantages of preserving enzyme activity and the extension of blood circulation time by avoiding the destruction by the mononuclear phagocyte system [27, 28]. As entering tumor tissues, the acidic microenvironment induced the decomposition of the liposomes, leading to exposure of the PEI-elastase/PD-L1 siRNA. Subsequently, PEI-elastase showed elevating catalytic activity and stability under acidified conditions, which in turn induced tumor killing and powerful tumor immunogenicity. Meanwhile, PEI-elastase facilitated PD-L1siRNA entry into tumor cells via NRP1-mediated transcytosis and PEI-mediated endocytosis, and then reduced tumor PD-L1 expression. With the synergism of the above aspects, P-E/S Lip greatly inhibited primary tumor growth and induced a systemic anti-tumor immune response, suppressing metastasis and distant tumors (Scheme. 1b). Since proteases have multiple biological activities, further, based on the nanocarriers developed in this study, we expect to explore more drug delivery systems that can be used in combination with nucleic acids or other bioactive agents to target tumors with minimal adverse side effects.

## Results

### Synthesis and characterization of PEI-elastase

**Fig. 1:**
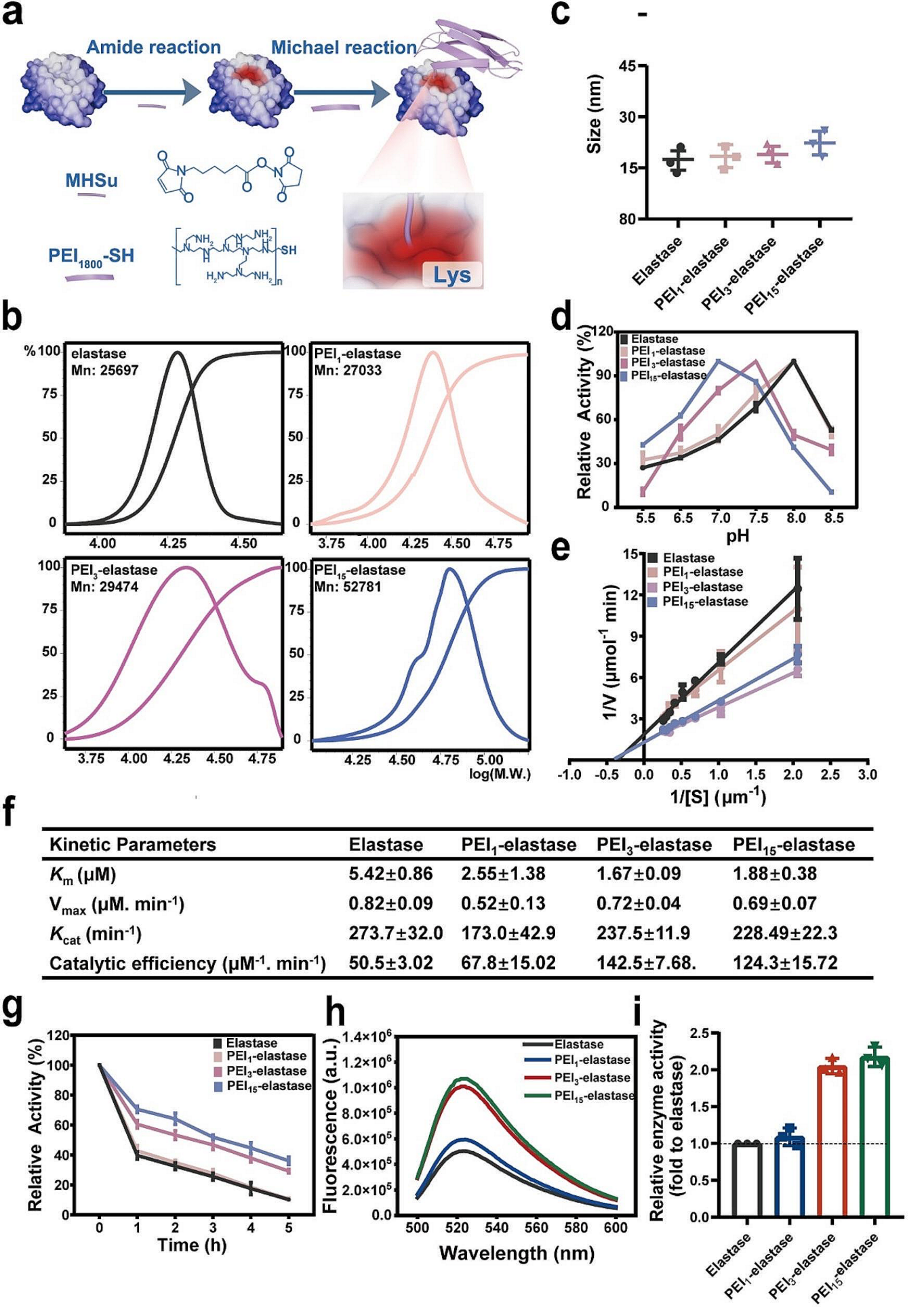
Preparation and characterization of PEI-elastase. (**a**) Schematic diagram of the synthetic route of PEI-elastase. (**b**) The molecular weights of elastase, PEI_1_-elastase, PEI_3_-elastase and PEI_15_-elastase measured by GPC. (**c**) The particle sizes of elastase, PEI_1_-elastase, PEI_3_-elastase and PEI_15_-elastase prepared at varied elastase/MHsu/PEI ratios (w/w), as measured by DLS. (**d**) pH-dependent activity curves of elastase, PEI_1_-elastase, PEI_3_-elastase and PEI_15_-elastase. Casein was utilized as a substrate with pH ranges of 5.5 to 8.5. (**e-f**) Michaelis-Menten curve for the determination of *Km*, *Vmax*, and *Kcat/Km* values, with casein concentrations plotted on the x axis and velocity plotted on the y axis. (**g**) Residual activities of elastase, PEI_1_-elastase, PEI_3_-elastase and PEI_15_-elastase after incubation with various time at pH 6.5 for the acid stability determinations. (**h**) The fluorescence intensity curves of fluorescence-quenched peptides after incubation with elastase, PEI_1_-elastase, PEI_3_-elastase or PEI_15_-elastase at pH 6.5. Fluorochrome in this peptide is initially quenched, and cleavage of the scaffold by elastase releases fluorochromes and generates extensive fluorescence. Higher fluorescence signal indicates more substrate cleavage by elastase enzyme activity. (**i**) Quantitative analysis of the relative enzyme activities of elastase, PEI_1_-elastase, PEI_3_-elastase or PEI_15_-elastase at pH 6.5, which were shown as the fold change from that of free elastase

PEI-elastase was synthesized via a Michael addition reaction and an amide coupling reaction mediated by a linker, 6-maleimidohexanoic acid N-hydroxysuccinimide ester (MHSu) (Fig. 1a). MHSu is a heterobifunctional reagent that crosslinks between amino and sulfhydryl groups. It has been used as a unique and useful reagent for preparing hapten conjugates and enzyme immunoconjugates [29]. In the conjugation process, MHSu was coupled initially to elastase through an amide bond (mainly lysine), followed by specific coupling with SH-modified PEI via maleimide at the end of the crosslinking agent MHSu. Given that chemical modification would be predicted to abolish the enzymatic activity of elastase, we first chose a 1:1:1 elastase to MHsu to PEI_1800_ low molar ratio for the fabrication of PEI-elastase. A 1:3:3 elastase to MHsu to PEI_1800_ molar ratio (three lysines in elastase) and a 1:15:15 elastase to MHsu to PEI_1800_ high molar ratio were also used for fabrication of PEI-elastase. As shown in Fig. 1b, Gel permeation chromatography (GPC) revealed that the average molecular masses of PEI-elastase increased with the elastase/ MHsu/ PEI ratio. Elastase had an average molecular mass of 25.7 kDa, whereas the average molecular masses for PEI_1_-elastase, PEI_3_-elastase and PEI_15_-elastase were 27.0 kDa, 29.5 kDa and 52.8 kDa, respectively. Dynamic light scattering (DLS) showed that elastase had an average particle diameter of 17.53 nm, while particle diameters for PEI_1_-elastase, PEI_3_-elastase and PEI_15_-elastase were 18.16 nm, 19.63 nm and 22.78 nm, respectively (Fig. 1c). The above results indicated that PEI molecules have linked onto the enzyme molecules after chemical modification, and the elastase modification with PEI at different molar ratios had been successfully achieved.

PEI_1_-elastase, PEI_3_-elastase and PEI_15_-elastase were investigated by examining the amount of tyrosine produced by enzymatic cleavage to probe the effect of PEI binding on elastase kinase dynamics. First, the optimum temperature for the enzyme reaction was determined. The activity% of elastase, PEI_1_-elastase, PEI_3_-elastase and PEI_15_-elastase were compared at pH 7.5 and the obtained higher enzymatic activity was assumed as 100% for each reaction. In Fig. S1a, it was found that elastase and PEI_1_-elastases exhibited maximum activity at 30 °C, but PEI_3_-elastase and PEI_15_-elastase showed maximum activity at 35 °C, respectively. Then, the temperature profile showed that, under the assay conditions used, both the elastase and PEI_1_-elastase showed similar pH optimum of 8.0 (Fig. 1d). However, the optimum pH for PEI_3_-elastase and PEI_15_-elastase shifted from pH 8.0 for elastase to pH 7.5 and pH 7.0, indicating that PEI-elastase may show better performance than free elastase in the acidic tumor microenvironment. Next, Michaelis–Menten model parameters were obtained with a graphical analysis using a Lineweaver–Burk plot (Fig. 1e and Fig. S1b) with the results summarized in Fig. 1f. The *Km* values the measure of affinity of enzyme to substrate. Meanwhile, the catalytic constant (*kcat*) can be obtained by using the equation of *kcat* = *Vmax*/[enzyme], and the catalytic efficiency can be evaluated by using the value of *kcat*/*Km*. Usually, the lower *Km* value while the higher *Vmax*, *kcat*, and *kcat*/*Km* values indicate the stronger enzymatic activity of enzymes [30]. As can be seen, the *Km*, *Vmax* and catalytic efficiency of free elastase were 5.42 ± 0.86 μm, 0.82 ± 0.09 μm/min and 50.5 ± 3.02 μm^− 1^ min^− 1^, respectively. Obviously, elastase modification by PEI decreased Km values and increased catalytic efficiency, suggesting conformational changes to the active form of elastase with enhanced affinity to the substrate. By comparing the kinetic parameters of PEI_1_-elastase, PEI_3_-elastase and PEI_15_-elastase, it was clearly seen that PEI_3_-elastase displayed the best enzymatic activity, followed by PEI_15_-elastase.

Given that elastase needs to play a role in the acidic tumor microenvironment, we checked the acid stability of the elastase, PEI_1_-elastase, PEI_3_-elastase and PEI_15_-elastase. The residual enzyme activities of free and modified elastases with various incubation time under pH 6.5 were shown in Fig. 1g. After incubation for 1 h, the residual relative activities of elastase and PEI_1_-elastase were remarkably reduced to about 40%, whereas the PEI_3_-elastase and PEI_15_-elastase retained about 60% and 70%, respectively. After, elastase and PEI_1_-elastase showed a constant reduction in enzymatic activity with the activity reduced to less than 10%. While PEI_3_-elastase and PEI_15_-elastase retained about 40%. Furthermore, we also used a special fluorescence-quenched peptide as a substrate to verify the above results. Fluorochrome in this peptide is initially quenched, but cleavage of the scaffold by elastase releases fluorochromes and generates extensive fluorescence [31]. Higher fluorescence signal indicates more substrate cleavage by elastase enzyme activity. The results showed that PEI_3_-elastase and PEI_15_-elastase possessed strong enzymatic activity, which had 2-fold greater activity than elastase and PEI_1_-elastase at pH 6.5. Moreover, PEI_15_-elastase exhibited slightly stronger enzymatic activity than PEI_3_-elastase (Fig. 1h and i). These above results indicated that chemical modifications of elastase by PEI improved elastase activity and acid stability. Of those, PEI_3_-elastase and PEI_15_-elastase performed comparably at pH 6.5. Considering that an excellent delivery capability of PEI-elastase is critical for design anti-tumor strategy in this study, we selected PEI_15_-elastase for subsequent experiments. By the way, we characterized the structural features of PEI_15_-elastase using three-dimensional fluorescence spectra and Circular dichroism (CD) spectroscopy. The characterization results indicated that this modification only slightly affected the secondary structures of elastase (Fig. S2).

### Gene-delivery efficacy of PEI-elastase

**Fig. 2:**
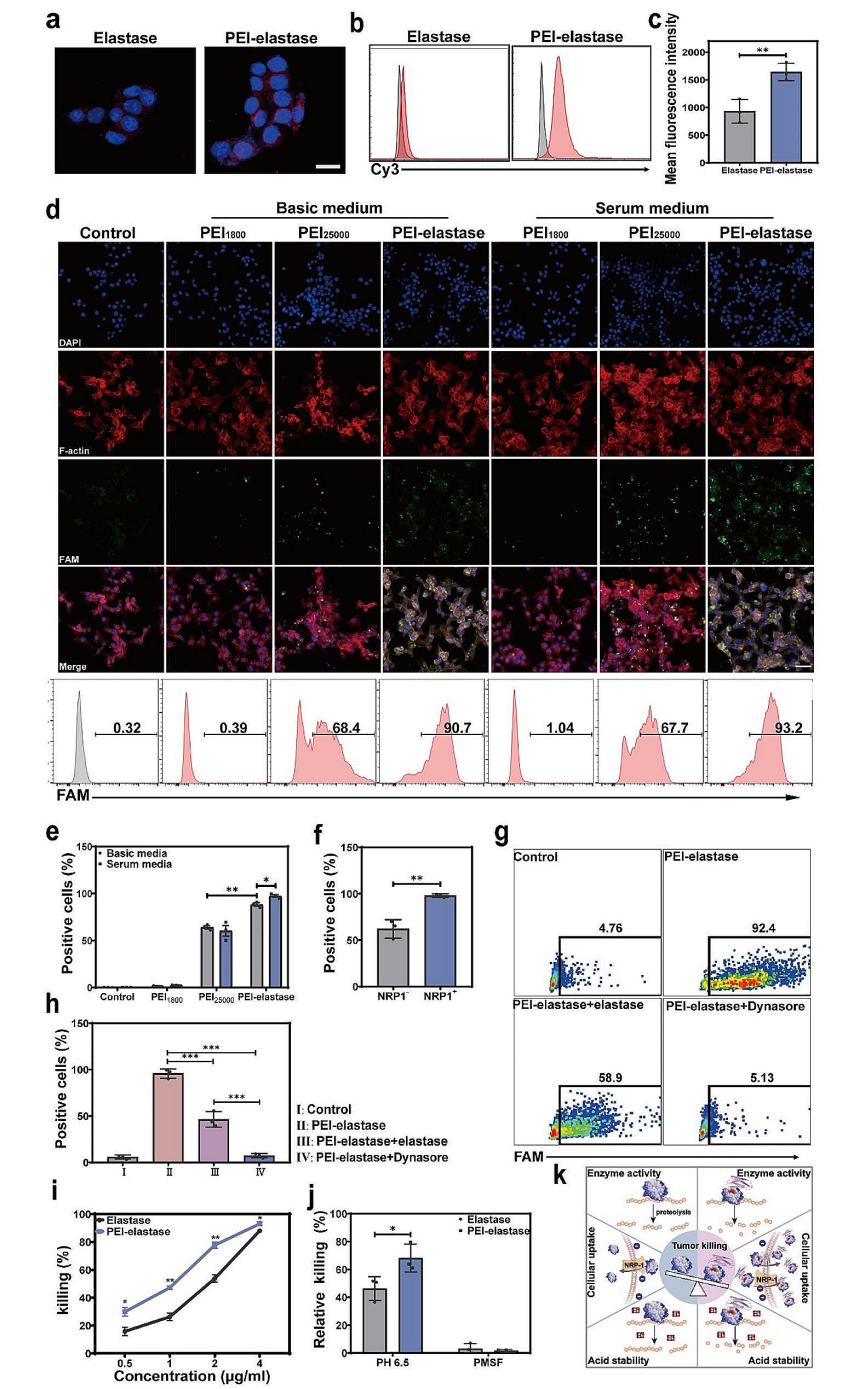
Gene delivery efficiency of PEI-elastase as a gene carrier. (**a**) CLSM images of cellular uptake of Cy3-labeled elastase and PEI-elastase in MDA-MB-231 cells (red = elastase or PEI-elastase, blue = nuclei; scale bars: 5 μm). (**b-c**) Flow cytometric analysis of the cellular uptake of Cy3-labeled elastase and PEI-elastase in MDA-MB-231 cells. (**d**) CLSM images of the internalization of FAM-labeled nucleic acid (green) after incubation with different delivery systems for 2 h (above). Cell cytoskeleton F-actin and cell nuclei were counterstained with rhodamine phalloidin (red) and 4,6-diamino-2-phenyl indole (DAPI) (blue), respectively. The scale bars: 50 μm. Flow cytometry analysis of the cells after incubated with different delivery systems carrying FAM-labeled nucleic acid for 2 h, respectively (below). (**e**) Flow cytometric quantitative analysis of cellular uptake efficiency of FAM-labeled nucleic acid with different delivery systems. (**f**) Flow cytometric quantitative analysis of NRP1 and FAM expression on MDA-MB-231 cells after incubated with PEI-elastase/FAM-DNA for 2 h. respectively. (**g-h**) Flow cytometry analysis of cellular uptake of FAM-labeled nucleic acid under different conditions. Tumor cells were treated with elastase or dynasore for 30 min before incubated with PEI-elastase/FAM-DNA. (**i**) Cytotoxic effects of elastase and PEI-elastase under various concentrations on MDA-MB-231 cells using Calcein AM dye release assay after 6 h incubation. (**j**) Killing efficiency of elastase and PEI-elastase for MDA-MB-231 cells at pH 6.5 or in the presence of PMSF using Calcein AM dye release assay. (**k**) Schematic illustration of the mechanism of enhanced anti-tumor efficacy of PEI-elastase. Data are presented as mean ± s.d. from three independent experiments (*n* = 3). *P* values were determined by unpaired two-tailed Student’s t-test (**c**,** e**,** f**,** i**,** g**), or one-way ANOVA with Tukey test (**h**). *p < 0.05, **p < 0.01, ***p < 0.001

It has been demonstrated that crosslinking low molecular weight PEI with biocompatible components could improve their ability to mediate the transmembrane delivery of genes into the cell cytosol, while promoting cellular uptake of the biocompatible components. Considering the positive correlation between NE uptake in tumors and killing efficiency, we first analyzed the impact of PEI modification on the cellular uptake of fluorescein-labeled elastase. Compared with elastase with a medium cellular uptake efficiency, PEI-elastase showed markedly greater intracellular fluorescence intensity within 2 h (Fig. 2a and c). These results indicate that PEI modification enhanced the cellular uptake of the elastase.

The potential of PEI-elastase facilitating the delivery of FAM-labeled DNA was tested to examine the utility of PEI-elastase in the delivery of nucleic acids. PEI_25000_ and PEI_1800_ were also employed in this study for comparison. Results were observed directly with confocal laser-scanning microscopy (CLSM). As shown in Fig. 2d, the apparent uptake of FAM-labeled DNA was observed from MDA-MB-231 cells incubated with PEI-elastase in basal and serum media. In contrast, much lower uptake levels were observed in the cells treated with PEI_25000_, while uptake was absent in those cells treated with PEI_1800_. These observations were also confirmed with flow cytometry analysis. Quantitative analysis of the flow cytometry results indicated that the uptake ratios in cells treated with PEI_25000_ in different medium were 50–60%. In contrast, uptake rates were above 90% in the PEI-elastase-treated cells. Moreover, it was unexpected that PEI-elastase induced higher delivery efficiency in the presence of serum than that in the serum-free medium (Fig. 2d and e).

To explore the mechanism underlying this effect, we first assessed whether the gene-delivery capacity of PEI-elastase was associated with NRP1, which had previously been shown to mediate elastase uptake by cancer cells. Flow cytometry showed that more FAM-labeled DNA was taken up in NRP1 positive cells than in NRP1 negative cells. Furthermore, the uptake ratio in NRP1 positive cells was above 90%, whereas the uptake rate was 60% in NRP1 negative cells (Fig. 2f and Fig. S3a). To further test the specificity of NRP1-dependent gene delivery, we developed free elastase to functionally block the extracellular binding domain of NRP1. Subsequent results showed that elastase partially blocked PEI-elastase/DNA internalization, and the uptake ratio decreased from 90% to about 50% (Fig. 2g). We then investigated the endocytosis pathway for the PEI-elastase/DNA using dynasore, a dynamin inhibitor of clathrin-mediated endocytosis. We observed that this inhibitor completely blocked this delivery pathway (Fig. 2g and h). These studies indicated that PEI-elastase could facilitate nucleic acid attachment and cell entry via NRP1-dependent endocytosis and crosslinked PEI-mediated endocytosis. In addition, we found that some nucleic acid delivered by PEI-elastase was not colocalized with lysosomes at 4 h, and their Pearson’s correlation coefficient was calculated to be 0.67, indicating that the complexes were endocytosed into the cells by the lysosomal pathway and could partially escape the lysosomes (Fig. S3b, S3c).

Upon observing the high enzyme activity and effective cellular uptake of PEI-elastase, we then investigated its tumor-killing activity, which has shown anti-cancer properties regarding cellular uptake and catalytic activity. Results showed a significant potentiation of tumor cell killing at increasing concentrations of the PEI-elastase(Fig. 2i). Furthermore, killing efficiency was notably higher at all PEI-elastase concentrations than that in elastase. In addition, the PEI-elastase remained above 70% relative to maximum killing activity under weak acid conditions, while elastase kept relative maximum killing rates below 50% (Fig. 2j). Notably, phenylmethylsulfonyl fluoride (PMSF, a serine protease inhibitor)-treated PEI-elastase could not kill tumor cells, suggesting no overt toxicity was associated with PEI and the linker (Fig. 2j).

As mentioned above, PEI-elastase could be uptake by NRP1 negative tumor cells, suggesting that PEI-elastase avoided the restrictions of NRP1-mediated endocytosis and achieved NRP1 negative tumor cell-killing ability. Thus, PEI-elastase enhanced the killing effect and expanded the window for the selective killing of cancer cells. In all, chemical modification of elastase with PEI improved the activity and acid stability, facilitating rapid cellular uptake and synergistically enhancing the elastase-mediated tumor killing (Fig. 2k).

### Synthesis and functional validation of P-E/S lip in vitro

**Fig. 3:**
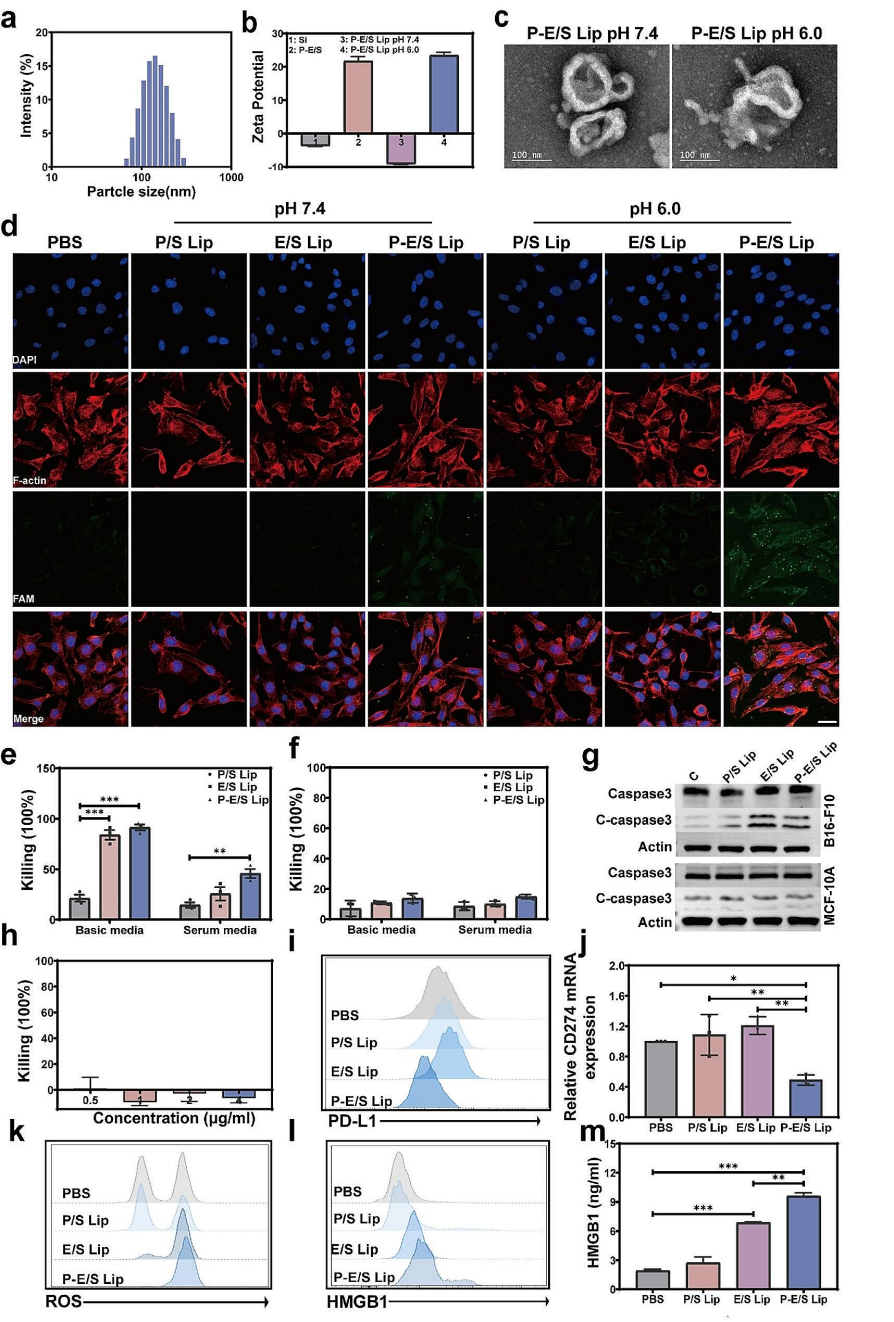
Synthesis and cytotoxicity of P-E/S Lip. (**a**) Size distribution of P-E/S Lip determined by DLS. (**b**) Zeta potential distributions of different species at different conditions. (**c**) TEM images of P-E/S Lip at pH 6.0 for 0 and 24 h, scale bar: 100 nm. (**d**) CLSM images of the internalization of FAM-labeled PD-L1 siRNA (green) after 2 h incubation with different liposomes. Cell cytoskeleton F-actin and cell nuclei were counterstained with rhodamine phalloidin (red) and 4,6-diamino-2-phenyl indole (DAPI) (blue), respectively. The scale bars: 20 μm. (**e-f**) Cytotoxic activity of different liposomes against B16-F10 cells (e) and MCF-10 A (f) after treatment at pH 6.5 for 6 h. The killing activity was assessed using Calcein AM release assay. (**g**) Western blot analysis of the Caspase3 and cCaspase3 expressions in B16-F10 cells and MCF-10 A cells after treating with different liposomes at pH 6.5 for 4 h. Actin was used as a loading control. **(h**) Cytotoxic activity of P-E/S Lip against mouse lymphocytes at various concentrations assessed by Calcein AM release assay. (**i**) Flow cytometry analysis of PD-L1 expression in B16-F10 cells after incubation with different liposomes for 3 days. (**j**) Levels of CD274 mRNA in B16-F10 cells after incubation with different liposomes at pH 6.5 for 2 days. (**k**) Flow cytometry analysis of ROS expression in B16-F10 cells after incubation with different liposomes at pH 6.5 for 2 h. (**l**) Flow cytometry analysis of CRT expression in B16-F10 cells after incubation with different liposomes at pH 6.5 for 24 h. (**m**) Enzyme-linked immunosorbent assay (ELISA) analysis of HMGB1 expression in the B16-F10 cell culture supernatants after incubation with different liposomes at pH 6.5 for 2 days. When cell experiments lasted more than 6 h under acid culture condition, the medium was removed and replaced by fresh media. Data are presented as mean ± s.d. from three independent experiments (*n* = 3). *P* values were determined by one-way ANOVA with Tukey test. *p < 0.05, **p < 0.01, ***p < 0.001

The above experiments demonstrated that PEI-elastase had high anti-tumor activities and excellent gene-delivery capabilities. Based on these findings, we developed a synergetic anti-tumor strategy through the synthesis of PEG-modified anionic liposomes for encapsulating PEI-elastase and PD-L1 siRNA (P-E/S Lip). First, the PEI-elastase/PD-L1 siRNA polyplex (P-E/S) core was developed through electrostatic interactions between positively charged PEI and negatively charged nucleic acids. Then, a layer of pH-responsive liposomes was coated onto the P-E/S with a negatively charged surface. The CLSM images presented the homogeneous distribution of elastase (Cy3) and PD-L1siRNA (FAM) in the liposomes (Cy5), indicating the successful wrapping of PEI-elastase/PD-L1 siRNA by liposome membrane (Fig. S4). DLS measurements revealed that the average particle size of the P-E/S Lip was 175.7 ± 10.2 nm (Fig. 3a). Further characterization indicated that the P-E/S had a positively charged surface with a zeta potential of + 21.7 ± 1.8 mV. In comparison, the potential zeta of the coated liposome declined to -9.15 ± 1.6 mV (Fig. 3b). Further zeta potential analysis indicated that P-E/S Lip presented a noticeable shift from − 9.15 to + 23.5 mV over 2 h when the pH changed from 7.4 to 6.0. This suggests the disassembly of the shell from liposomes and the exposure of the polyplex core. The morphology of P-E/S Lip was detected using transmission electron microscopy (TEM), which showed that liposomes were formed as discrete spherical vesicles with sizes around 100 nm. And the spherical particles contained the content like P-E/S polyplex. The PEG layer protected liposomes from aggregation. Next, we evaluated the responsiveness of P-E/S Lip to acidic environments. The TEM measurements showed that there are irregular particles with partial membrane fusion in the visual field (Fig. 3c). Considering the different pH values in the bloodstream (pH 7.4) and tumor microenvironments (pH 5.5 to 6.5), this responsive shell structure allows the P-E/S Lip to present different surfaces with the desired physical and chemical properties. This is suggested as the best possibility for liposomes to overcome physiological barriers and efficiently deliver PEI-elastase/PD-L1 siRNA to tumor tissue.

We assessed the potential of P-E/S Lip to promote its cellular uptake and thereby increase PEI-elastase and PD-L1 siRNA accumulation in cells. After preparing different liposomes (FAM-labeled siRNA) and then incubating them with MDA-MB-231 cells, the cellular uptake behaviors of PD-L1 siRNA were observed with CLSM and flow cytometry. As shown in Fig. 3d and Fig. S5a, very low level of FAM fluorescence was observed in cells treated with elastase/PD-L1 siRNA liposome (E/S Lip) and PEI_1800_/PD-L1isRNA liposome (P/S Lip) at pH 6.0 and pH 7.4. These results were attributed to the inefficient cellular uptake of the PD-1siRNA. In contrast, high-level fluorescence expression was observed in the cells treated with P-E/S Lip at pH 6.0.

After documenting the effective cellular uptake and delivery of P-E/S Lip, we next verified the ability of P-E/S Lip to induce tumor cell death. B16-F10, MDA-MB-231, and MCF-10 A cells were separately incubated with P-E/S Lip, E/S Lip, or P/S Lip at pH values 6.5 or 7.4, respectively. The killing effects were measured using a calcein AM dye release assay after 6 h of incubation. B16-F10 and MDA-MB-231 cells exhibited low viability after incubation with P-E/S Lip and E/S Lip. P-E/S Lip showed more efficient in killing tumor cells than E/S Lip group in a serum medium at pH 6.5 (Fig. 3e and Fig. S5b). This could be explained by the high catalytic efficiency and cellular uptake efficiency of PEI-elastase. In contrast, none of these groups showed obvious toxic to non-cancerous MCF-10 A cells (Fig. 3f). Tumor cell apoptosis was also verified with flow cytometry and CLSM. As shown in Fig. S5c-S5d, early and late apoptosis of P-E/S Lip-treated cells were significantly increased compared with early and late apoptosis of other groups treated cells at pH 6.5. In contrast, there was no significant difference in observed apoptosis among these groups at pH 7.4. We also determined the level of the apoptosis–related protein caspase-3 to confirm the involvement of apoptosis. Activation of effectors of apoptosis (increased cleaved caspase-3) was notable in the P-E/S Lip and E/S Lip-treated B16-F10 cells at pH 6.5 (Fig. 3g). In addition, we further detected whether P-E/S Lip was toxic to immune cells. Results showed that two groups demonstrated minimal killing activity of human and mouse lymphocytes at all tested concentrations, confirming the targetability and security of PEI-elastase-based liposomes (Fig. 3h and Fig. S5e). These results suggested that P-E/S Lip could effectively and specifically induce tumor cell apoptosis in a pH-dependent manner.

After demonstrating the effective cellular uptake of P-E/S Lip, we next sought to determine whether siRNA can perform its biological function of regulating gene expression. We chose CD274 (PD-L1) in B16-F10 cells as a target for silencing, and PD-L1 overexpression was demonstrated by flow cytometry (Fig. S5f). Results showed that PD-L1 mRNA and protein were downregulated in a pH-dependent manner in the P-E/S Lip group (Fig. 3i and j). This suggested that P-E/S Lip could efficiently deliver siRNA into cancer cells to knock down the genes of interest. Notably, E/S Lip treatment further upregulated PD-L1 expression in tumor cells.

Elastase was also previously reported to induce CD8^+^ T cell-mediated anti-tumor response, which suggested that PEI-elastase may potentially induce immunogenic cell death (ICD) in the tumor. To test this hypothesis, we looked for the hallmarks of ICD in different liposomes-treated tumor cells in vitro. We detected increased tumor cell reactive oxygen species (ROS) expression after treatment with E/S Lip and P-E/S Lip (Fig. 3k and Fig. S6a). Meanwhile, we observed an increased secretion of high-mobility group protein 1 (HMGB1) in the culture supernatant and expression of calreticulin (CRT) on the tumor cell surface after treatment with E/S Lip and P-E/S Lip (Fig. 3l and m and Fig. S6b). Meanwhile, ICD was considerably stronger in the P-E/S Lip-treated tumor than in the E/S Lip-treated cells. Taken together, we demonstrated that P-E/S Lip can induce ICD upon specific tumor cell killing and regulate the expression of tumor PD-L1.

### Anti-tumor efficacies of intratumoral administration of P-E/S lip

**Fig. 4:**
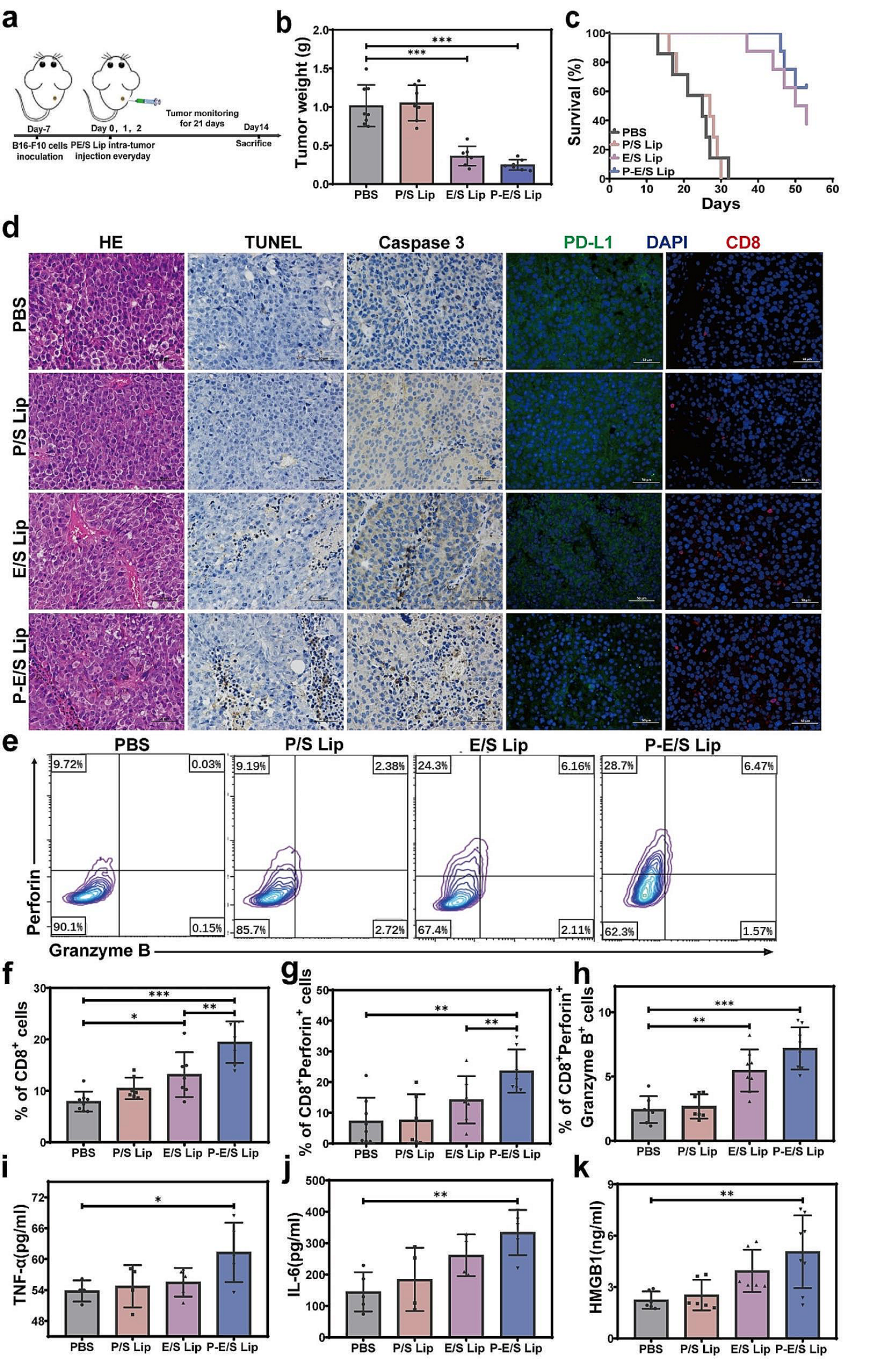
Anti-tumor activities of P-E/S Lip via intratumoral administration. (**a**) Schematic time-line of in vivo anti-tumor experiments with intratumoral administration. (**b**) Average weight of tumors collected after various treatments for 14 days, (*n* = 4–8). (**c**) Survival time of the mice in various groups displayed as Kaplan-Meier curves (*n* = 4–8 mice/group). (**d**) HE staining, TUNEL staining and Caspase 3 assay of the tumor tissues in each treatment group (left). IF staining images analysis of CD8 and PD-L1 expression in primary tumor sections from mice following various treatments (right). Scale bar: 50 μm. (**e-h**) Representative flow cytometric plots and quantification of functional CD8^+^ T subsets in primary tumors at day 14 after different treatments. (**i-j**) ELISA analyses of expression levels of IL-6 and TNF-α within B16-F10 tumor tissues in each treatment group. (**k**) ELISA analyses of expression levels of HMGB1 within B16-F10 tumor tissues in each treatment group. Data are presented as mean ± s.d. *p* values were determined by one-way ANOVA with Tukey test. *p < 0.05, **p < 0.01, ***p < 0.001

Based on the efficient tumor killing and PD-L1 downregulation by P-E/S Lip in vitro, we explored whether the liposomes effectively inhibit tumor growth in vivo. A subcutaneous tumor model was established using B16-F10 cells and treated with different liposomes via intratumoral administration (once a day for three days). Tumor growth was measured every two days (Fig. 4a). Compared with the control, P/S Lip exhibited little anti-tumor effect. In contrast, E/S Lip and P-E/S Lip treatment greatly inhibited tumor growth, and the tumor size of P-E/S Lip-treated mice was roughly one-fifth the size of those receiving PBS treatment (Fig. 4b and Fig. S7a-S7b). Consistently, survival curves showed that mice treated with P-E/S Lip and E/S Lip had markedly prolonged survival times. Meanwhile, P-E/S Lip improved therapeutic efficacy by extending survival time compared with E/S Lip (Fig. 4c). Then, hematoxylin (HE) and eosin staining, terminal deoxynucleotidyl transferase dUTP nick end labeling (TUNEL), and caspase-3 staining of the tumor tissues showed more signs of apoptosis in the P-E/S Lip-treated tumor than in the other groups (Fig. 4d).

To evaluate the induction of anti-tumor immune responses by P-E/S Lip, we studied the proportion of immune cells in the tumor tissues after various treatments using immunofluorescence (IF) and flow cytometry. IF staining showed that PD-L1 protein expression was significantly reduced upon P-E/S Lip treatment, and there were more infiltration of CD8^+^ T cells in the P-E/S Lip-injected tumors than that in the control groups (Fig. 4d). Moreover, flow cytometric analyses showed that, among CD8^+^ T subsets, P-E/S Lip-mediated immunotherapy elicited 24% of CD8^+^ perforin^+^ T cells and 7.8% of perforin^+^ granzyme B^+^ T cells. These percentages were greater than those for the E/S Lip group (15.7% and 5.8%, respectively), the P/S Lip group (8.0% and 2.8%, respectively), and the PBS group (7.8% and 2.7%, respectively) (Fig. 4e and h). We also detected immune-related cytokines in tumor tissues by ELISA. The tumor treated with P-E/S Lip expressed higher protein levels of TNF-α and IL-6 compared with the control tumors (Fig. 4i and j). Finally, we validated the ICD biomarkers in the different liposomes-treated tumor tissues. HMGB1 significant increase in tumor was found in the E/S Lip and P-E/S Lip groups compared with the control group (Fig. 4k). Immunohistochemistry showed increased expression of CRT in the E/S Lip and P-E/S Lip-treated tumors (Fig. S7c). These findings demonstrated that P-E/S Lip elicited direct tumor cell killing and significant anti-tumor immune responses in situ via the induction of ICD and the downregulation of PD-L1 expression.

### Systemic anti-tumor immune responses induced by P-E/S lip

**Fig. 5:**
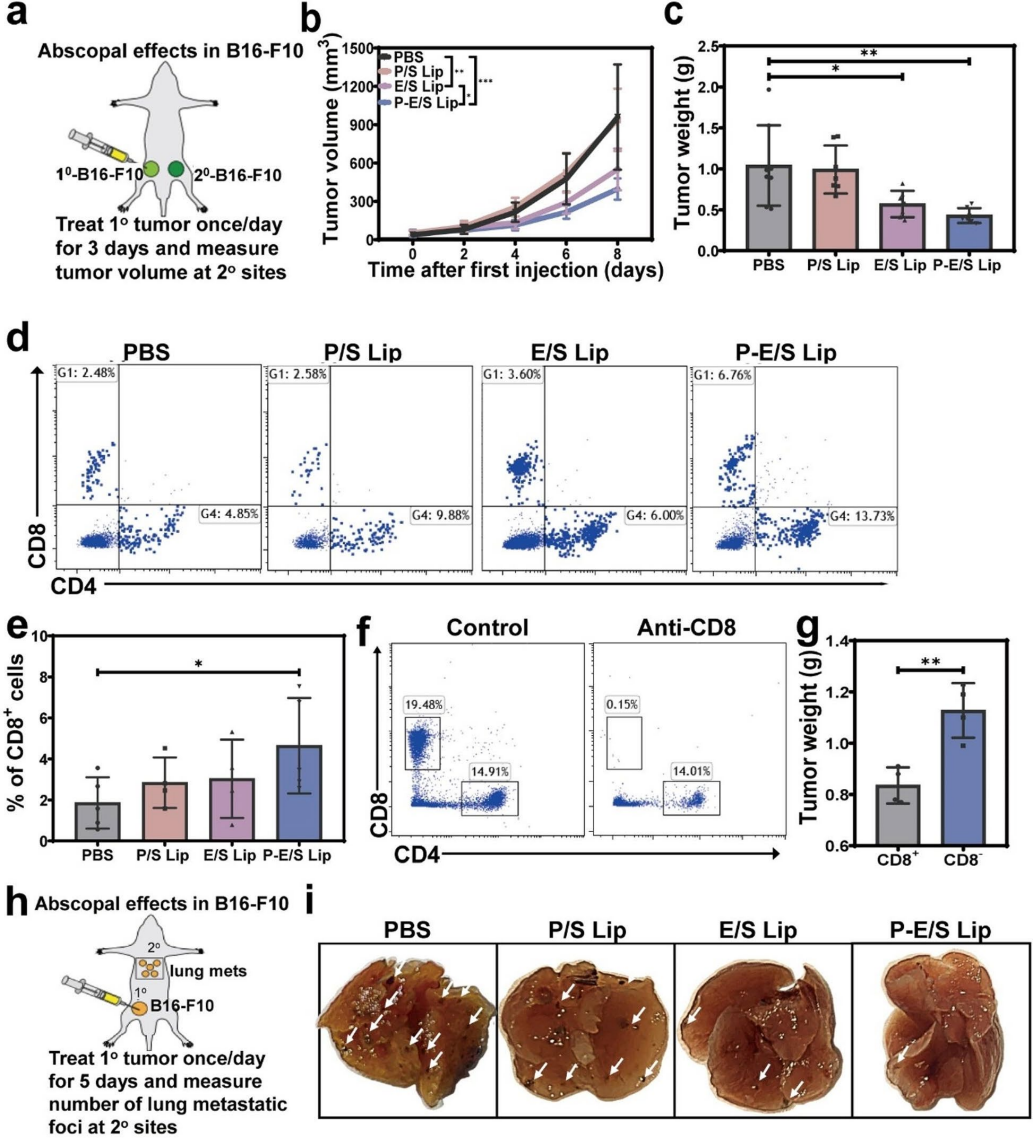
Evaluation of P-E/S Lip-mediated systemic anti-tumor immune response. (**a**) Schematic for testing the P-E/S Lip-mediated abscopal effect. (**b**) Curves of tumor growth at distant sites in B16-F10 tumor-bearing mice after primary tumor treatments for 3 days. (**c**) Average weight of tumors at distant sites collected after various treatments for 14 days, *n* = 5. (**d**,** e**) Representative flow cytometric plots and quantitative analysis of CD8^+^ T cell subsets in tumors at distant sites at day 14 after treatment. (**f**) Validation of CD8^+^ T cell depletion with anti-CD8 antibodies. Quantification of % abundance in blood. (**g**) Effect of depleting CD8^+^ T cells on P-E/S Lip-mediated anti-tumor efficacy in non-injected tumors. (**h**) Schematic of the experimental process for lung metastasis model. (**i**) Whole-lung images of metastasis nodules at the lung surface on day 21 after orthotopic injection of B16-F10 cells (*n* = 5). Data are presented as mean ± s.d. *P* values were determined by unpaired two-tailed Student’s t-test (**g**), or one-way ANOVA with Tukey test (**b**,** c**,** e**). *p < 0.05, **p < 0.01, ***p < 0.001

Previous studies have shown that elastase uptake by cancer cells enhanced tumor immunogenicity to activate cytotoxic T cells, which decreased tumorigenesis at distant sites through CD8^+^ T cells, a property referred to as the abscopal effect. Thus, we investigated whether treating a primary tumor with P-E/S Lip could inhibit tumor growth at distant sites through the induction of systemic anti-tumor immune responses. To test this, a bilateral tumor model was established using B16-F10 cells, which involved treating one side of the tumor with different liposomes once a day for three days and measuring tumor growth at distant sites (Fig. 5a). Although the mean tumor size increased over time in all groups, the tumor growth volume and tumor size at distant sites in P-E/S Lip and E/S Lip groups were lower than those in PBS group (Fig. 5b and c). In addition, the volumes of tumors treated with P-E/S Lip were significantly lower than those of tumors in the E/S Lip group.

The tumor-infiltrating immune cells in tumor tissues at distant sites were examined by flow cytometry. Results showed that intratumoral CD8^+^ T cell densities were significantly higher in tumors with P-E/S Lip treatment compared with PBS treatments (Fig. 5d and e). Apart from these, no other notable differences were detected. To examine the dependence of this abscopal effect on CD8^+^ T cells, we repeated this experiment in mice where CD8^+^ T cells were depleted before and after P-E/S Lip treatment. Results showed that the depletion of CD8^+^ T cells attenuated the abscopal effect mediated by P-E/S Lip in tumor models (Fig. 5f and g).

Whether P-E/S Lip could induce a CD8^+^ T cell-mediated inhibition of tumor metastasis was further investigated. A melanoma model was established in which B16-F10 cells were injected into the flank for primary tumors and intravenously to create secondary lung tumors. In this model, we treated the primary tumor with different liposomes (once daily for five days), and the number of visible lung metastasis nodules was determined (Fig. 5h). Results showed that the numbers of metastatic foci were lower in the P-E/S Lip group compared with that the PBS group (Fig. 5i). The above results suggested that orthotopic tumor treatment with P-E/S Lip could trigger systemic anti-tumor immune responses, suppressing distant tumors and metastasis.

### Anti-tumor efficacies of intravenous administration of P-E/S lip

**Fig. 6:**
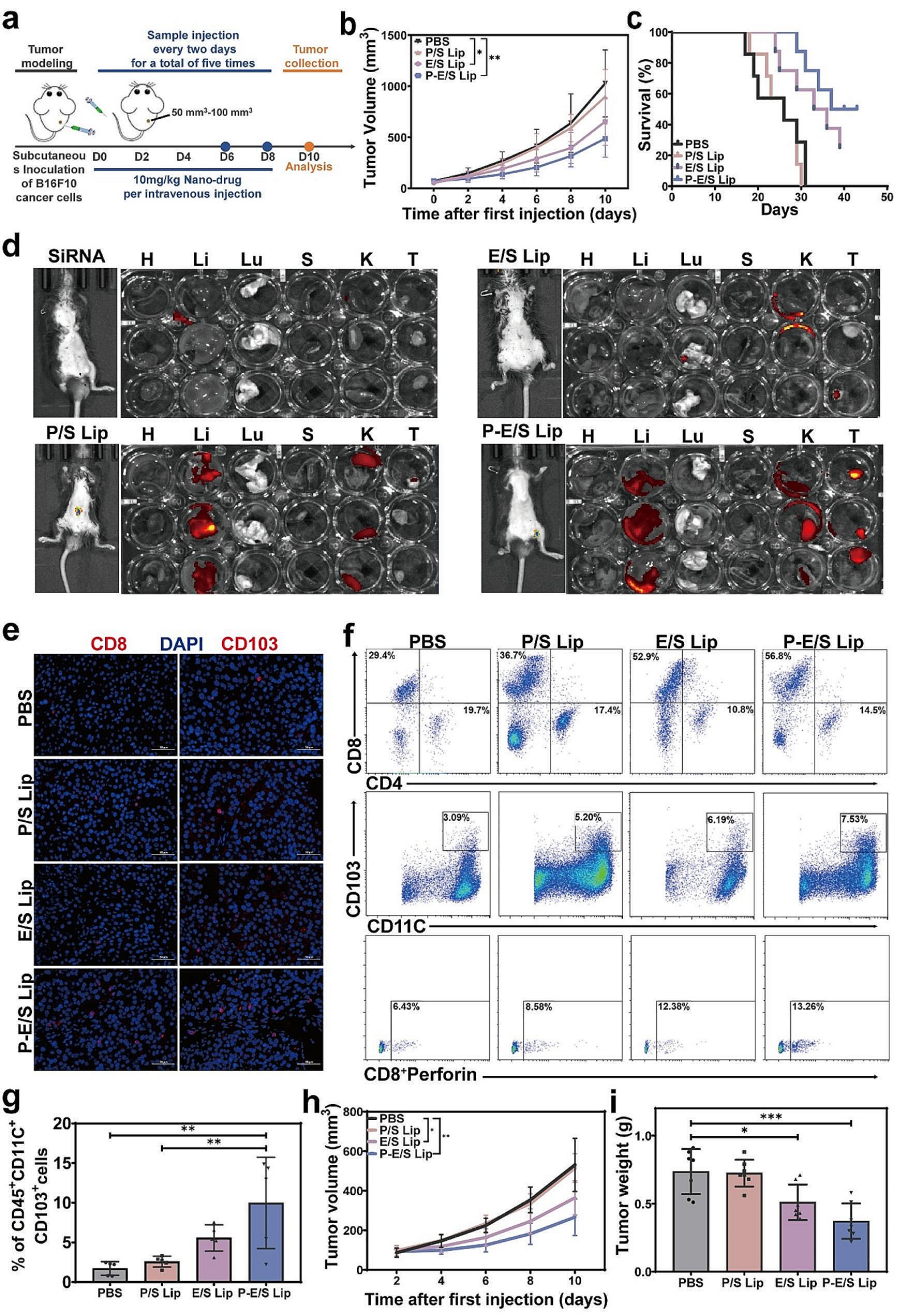
Anti-tumor efficiency of P-E/S Lip following intravenous administration. (**a**) Schematic illustration of anti-tumor experimental schedule with intravenous administration. (**b**) Curves of tumor growth in B16-F10 tumor-bearing mice after different treatments. **(c**) Survival time of the mice in various groups displayed as Kaplan-Meier curves (*n* = 4–7). (**d**) IVIS imaging whole-body of 4T1 tumor-bearing mice and ex vivo imaging of tumors and major organs at 24 h following intravenous administration of free Cy5.5-siRNA or various Cy5.5-siRNA liposomes (*n* = 3). H, heart; K, kidney; S, spleen; Lu, lung; Li, liver; T, tumor. (**e**) IF staining images analysis of CD8^+^ and CD103^+^ cells in primary tumor sections from mice following various treatments. Scale bar: 50 μm. (**f**,** g**) Representative flow cytometric plots and quantitative analysis of CD11c^+^CD103^+^ cell subsets in primary tumors at day 14 after treatment. (**h**) Curves of tumor growth in 4T1 tumor-bearing mice after different treatments, (*n* = 5). (**i**) Average weight of 4T1 tumors collected after various treatments for 14 days (*n* = 6–8). Data are presented as mean ± s.d. *P* values were determined by one-way ANOVA with Tukey test. *p < 0.05, **p < 0.01, ***p < 0.001

The encouraging anti-tumor effects of P-E/S Lip against B16-F10 xenograft tumors via intratumoral administration prompted us to evaluate the potential anti-tumor effect of the liposomes via intravenous administration. The B16-F10 cancer cell xenograft model was established by implanting B16-F10 cells stably expressing firefly luciferase (B16/F10-Luci) subcutaneously into C57BL/6 mice. After 7 days, each tumor-bearing mouse was assigned to one of four groups and injected intravenously with PBS, P/S Lip, E/S Lip, or P-E/S Lip. The formulations were administered five times (once every other day), and the tumor volumes were recorded and monitored by in vivo bioluminescence imaging (Fig. 6a). As shown in Fig. 6b and Fig. S**8**a, mice treated with P/S Lip did not exhibit any tumor growth inhibition compared with mice in the PBS group. In contrast, mice treated with E/S Lip exhibited partial tumor growth suppression. Notably, the P-E/S Lip group exhibited the most effective tumor inhibition among all the groups. The biophotonic imaging of tumors further confirmed the results. Luciferase expression levels in tumors treated with P-E/S Lip were significantly lower than those in tumors treated with E/S Lip and PBS (Fig. S8b). Survival curves showed that mice treated with P-E/S Lip and E/S Lip had markedly prolonged survival times (Fig. 6c). In particular, the P-E/S Lip group enhanced tumor inhibition compared with the E/S Lip group. This indicated that PEI modification-mediated enzyme activity enhancement and PD-L1 siRNA delivery improved the therapeutic efficacy. We also assessed biosafety after systemic administration of various liposomes. Results indicated that mouse body weights were generally stable during the dosing period, and histologic analysis data showed no apparent damage or lesions in major organs (Fig. S8c-S8d). These results indicated the apparent biosafety of anti-tumor treatment based on P-E/S Lip.

The biodistribution and tumor site accumulation of cyanine5.5-PD-L1 siRNA in vivo were investigated with an in vivo imaging system (IVIS) at 24 h following the intravenous injection of different liposomes. Cyanine-5.5 signals were barely detectable in tumors following injection of free cyanine-5.5-siRNA and E/S Lip. Elastase did not have enough positive charge density to form the complex with PD-L1siRNA. Therefore, E/S Lip-encapsulated PD-L1siRNA showed relatively less amount, resulting in not enough siRNA-derived fluorescence at tumor. The P/S Lip-treated mice showed detectable fluorescence intensity in the liver and kidney, and fluorescence signals primarily accumulated in the liver, kidney, and tumor in the P-E/S Lip group (Fig. 6d). The results suggested that PEI-elastase greatly improved the cyanine-5.5-siRNA accumulation at the tumor site, with a decrease in liver uptake compared with P/S Lip. In all, positively charged PEI-elastase complexation with siRNA facilitated siRNA loading into the anionic liposomes and enhanced the tumor uptake of cyanine-5.5-siRNA.

The proportion of immune cells in the tumors after various treatments using IF and flow cytometry was analysed to illustrate the anti-tumor immune response of the P-E/S Lip. Because dendritic cells (DCs) play a significant role in triggering CD8^+^ T cell immune response, we investigated the DCs and CD8^+^ T cell infiltration in the tumor tissues of mice that had received the various treatments. IF staining demonstrated the accumulation of tumor-infiltrating CD8^+^ and CD103^+^ cells in the tumor tissue sections of mice treated with P-E/S Lip (Fig. 6e). Flow cytometric analysis further confirmed that the percentage of DCs (CD11c^+^CD103^+^) increased significantly to 7.4% in the mice treated with P-E/S Lip, which was 2.4-fold and 1.7-fold higher than the percentages of those cells in the PBS and P/S Lip groups, respectively (Fig. 6f and g).

Lastly, the anti-tumor efficacy of P-E/S Lip on 4T1 tumor mouse models was evaluated in order to assess the broader applicability of our approach. because elastase inevitably faced serum protease inhibition after intravenous administration, despite the protection effect of the liposome shell. P-E/S Lip exhibited slightly better performance than control groups via intravenous administration, which was consistent with the B16-F10 tumor model results (Fig. 6h and i and Fig. S9a). Meanwhile, no obvious body weight change or histologic damage to the main organs was observed in any groups (Fig. S9b-S9c). Although the anti-tumor effect of P-E/S Lip in vivo was not as significant as those in vitro, due to the complex physiological environment. P-E/S Lip still retained anti-tumor advantage compared with the other agents, presenting an efficacious and safe anti-tumor strategy.

## Discussion

Current cancer immunotherapies mainly focus on the regulation or modification of immune cells. However, many tumors are less immunogenic and exhibit low T cell infiltrate, thereby leading to immune escape and failure of immunotherapy [3, 32]. In this study, we developed a novel elastase-based anti-tumor immunotherapeutic strategy. The main innovations of this paper can be concluded as the following two points. On the one hand, elastase activity and acid stability were significantly increased by modification with PEI. And the PEI-modified elastase was also identified as an excellent gene-delivery material. On the other hand, the prepared P-E/S Lip not only directly mediated tumor killing and improved tumor immunogenicity, but also facilitated tumor infiltration of CD8^+^ T cells and reduced tumor PD-L1 expression. With the synergism of the above several aspects, this strategy inhibited tumor growth and induced a systemic anti-tumor immune response, leading to suppression of the distant tumor and metastasis. This work was also indicated that multiple functions may be achieved by modifying a protease with PEI, which provided a strategy for future work with various proteases.

MHSu is a heterobifunctional reagent cross-linking between amino and sulfhydryl groups in cross-linking proteins [29]. The elastase was covalently bonded to SH-modified PEI via maleimide at the end of the crosslinking agent MHSu. PEI has a large number of amine groups, which can be cross-linked under native conditions using excess crosslinker in the system [33, 34]. This prediction was experimentally confirmed by our experimental finding. It was the self-crosslinking of PEI that maked PEI-elastase a useful approach for protein and gene delivery. Moreover, we have demonstrated that PEI-elastase could effectively delivery large nucleic acids (> 10,000 bp, data not shown). Predictably, other proteins or peptides can also be attempted with this method, which would contribute to development of personalized protein or gene delivery strategies.

Elastase is a serine protease, which active site is composed of the amino acid triad histidine-aspartate-serine [35]. Elastase utilize a uniquely activated serine residue in the substrate-binding pocket to catalytically hydrolyse peptide bonds, form a tetrahedral adduct with the serine residue of the catalytic triad. Then, the histidine acts as a general base during catalysis, accepting a proton from the serine as it forms a bond with the substrate carbonyl carbon, regulating the catalytic process along with aspartate [36]. Our experimental results indicated that the covalent modification of elastase with PEI substantially decreased the enzyme’s Km value. This result is likely because the lysine residues are close to the substrate-binding region from the analysis of elastase structure, which modification caused partial disruption of hydrogen bonds around substrate binding domain of elastase. Therefore, the structure of elastase became slightly loose, allowing the enzyme more accessible to the substrate and increasing substrate affinity. In addition, modification with PEI shifted the optimal pH of elastase reactivity from 8.0 to 7.0. This change in pH preference suggests that the tethers may affect the protonation of the leaving group at low(er) pH in addition to altering the enzymatic hydrolysis. The main objective is to improve the ability of the elastase to withstand the tumor microenvironment (pH 6.5) and endosome/lysosome microenvironment (pH 4.5–5.5). It is regretful that elastase structure was not further resolved due to the flexible structure and the unclear cross-linking sites of PEI. Thus, many details about the structure of this modified protease are still unknown and further study is needed.

Previous studies have confirmed that NE enters the cell by first binding to the NRP1 on the surface of tumors, and then interacts with specific domains to selectively eradicate cancer cells. It can be noticed that NRP1 negative tumor could evade NE-mediated killing. We have showed that PEI-elastase could lead to this protease uptake by NRP1 negative tumor cells. It implied that PEI-elastase broke the restrictions of receptor mediated endocytosis and achieved NRP1 negative tumor cell-killing ability. Moreover, PEI has been demonstrated to possess good immune adjuvant potential [37]. in our combination therapy, multiple factors acting in concert induced a robust anti-tumor immune response.

## Conclusions

In summary, our study provides an protease-based anti-tumor strategy to enhance tumor immunogenicity and reduce tumor PD-L1 expression, thus exerting synergistic anti-tumor activities. We first synthesized PEI-elastase and confirmed that PEI modification improved catalytic activity and stability of elastase under acidified condition. PEI-elastase also facilitated gene entry into tumor cells via NRP1-mediated transcytosis and PEI-mediated endocytosis. Based on the above results, we developed a synergetic anti-tumor strategy (P-E/S Lip). All in vitro and in vivo results confirmed that P-E/S Lip showed an excellent inhibit primary and metastatic tumor efficacy, presenting great prospects for clinical application.

## Materials and methods

### Synthesis of PEI-elastase

10 mg elastase was dissolved in PBS (pH 7.4), 1.2 μm 6-Maleimidohexanoic acid N-hydroxysuccinimide ester (MHSu) was dissolved in DMSO. Then mixed elastase with MHSu with a certain ratio, and the solution was rotated for 2 h at 25 ℃. Then, PEI_1800_-SH (6 thiol groups attached to each PEI, Ruixi, Xian, China) was added to the solution of elastase at a certain molar ratio and rotated end over end for 1 h at room temperature to allow for conjugation PEI-elastase. Lastly, the PEI-elastase was prepared for use before having its buffer exchanged with PBS by using dialysis (3.6 kDa MWCO) [38].

### Characterization of PEI-elastase

Fluorescence spectrum: the excitation wavelength was increased from 200 nm to 350 nm, and the emission wavelength was increased from 200 nm to 600 nm, each time by 5 nm; the scanning speed was 600 nm/min, and the excitation and emission slit widths were 15.0 nm. Circular dichroism: the scanning data in the range of 250 –190 nm were recorded; sweep speed 50 nm/min; response time 2 s; response wavelength width 1.0 nm.

### Catalytic function of PEI-elastase

For elastase activity assays, casein was used as substrate for reaction under different pH, and the absorbance of 680 nm was determined with Folin reagent as chromogenic reagent. The amount of tyrosine produced by the reaction was calculated according to the standard curve of tyrosine measured by the experiment. 1 µg tyrosine produced by hydrolyzing casein per minute was defined as 1 protease activity. BHQ-EPFWEDQ-FAM peptide was also used to evaluate the catalytic activity of elastase and PEI-elastase by a Cary Eclipse Fluorescence Spectrophotometer (Agilent Technologies, Palo Alto, CA) [39]. The Km value of the dynamic and mechanical parameters and the Vmax value of the maximum reaction velocity of the enzyme were calculated by Lineweaver–Burk curves.

### Fluorescence labeling of PEI-elastase

200 µl solution of PEI-elastase (2 mg/ml) in sodium bicarbonate buffer (100 µl) was prepared. Next, 9.14 µl of a Cy3-NHS solution in DMSO (10 mg/ml) were added, and the mixture was incubated for 4 h at 37 °C and shaking in the dark. The solution of labeled PEI-elastase was then dialyzed in PBS (3.5 kDa pore membrane) for 24 h to eliminate nonreacted Cy3 molecules [40].

### Cell culture

MDA-MB-231, B16-F10, B16-F10/Luc and 4T1 cells were cultured in RPMI 1640 supplemented with 10% (v/v) FBS in a 37 °C incubator under 5% CO_2_ and 90% humidity and maintained at densities between 5 × 10^5^ and 2 × 10^6^ cells/ml. MCF-10 A was cultured in special medium (CM-0525, Pricella, China.)

### Delivery ability of PEI-elastase

CLSM (Olympus, FV1000) was used to evaluate the gene delivery ability of PEI-elastase. Briefly, MDA-MB-231 cells were seeded at a density of 1 × 10^4^ cells per well in a 35 mm confocal dish and incubated overnight for cell attachment. When cells reached a confluence of 50%, 4 µg/ml PEI-elastase/DNA were added respectively, and then cells incubated in complete culture medium for 2 h. To compare the efficiency of the cell transfection, PEI_1800_ and PEI_25000_ (Beyotime Biotechnology, Shanghai, China) were used for control. After the incubation, the cells were washed with ice-cold PBS for 3 times and fixed with fresh 4% paraformaldehyde for 15 min at room temperature. The cells were further counterstained with DAPI(Beyotime Biotechnology, Shanghai, China)and F-actin respectively following the manufacturer`s instructions. After the staining and washing, the cells were imaged using CLSM.

### Flow cytometry

Cells were digested using trypsin and harvested by centrifugation at 1200 rpm for 5 min. For membrane protein staining, antibody was added to the collected cell solution at the concentration of 2 µg/ml and incubated for 15 min from light. For intracellular protein staining, cells were fixed for 15 min using fresh 4% paraformaldehyde at first, then permeabilized for another 5 min, antibody was added to the collected cell solution at the concentration of 2 µg/ml and incubated for 15 min from light. After washing with PBS buffer and centrifugation, flow cytometry (BECKMAN COULTER) was performed. Antibodies used in this work were as follows: Brilliant Violet 510™ anti-mouse CD45 (30-F11), Brilliant Violet 421™ anti-mouse CD11c (N418), PE anti-mouse CD274 (MIH7), PerCP/Cyanine5.5 anti-mouse CD4 (GK1.5), Alexa Fluor^®^ 700 anti-mouse CD8a (53 − 6.7), APC anti-human/mouse Granzyme B (QA16A02), PE anti-mouse Perforin (S16009B), Brilliant Violet 421™ anti-mouse CD279 (29 F.1A12), FITC anti-mouse CD69 (H1.2F3), Alexa Fluor^®^ 700 anti-mouse CD103 (2E7), APC anti-mouse/human CD11b (M1/70), PE anti-mouse IgG (Poly4053), FITC anti-mouse IgG (Poly4060), the above antibodies were purchased from BioLegend. PE anti-mouse NRP1(123,129, Yubo), FITC anti-mouse/human Calreticulin (bs-5913R, Bioss).

### Synthesis of P-E/S lip

PEI-elastase and PD-L1 siRNA were dissolved PBS at 6:1 ratio (m/m), and mixed for 30 min to form PEI-elastase /siRNA, P-E/S Lip were prepared by a thin-film hydration method. In brief, a mixture of DSPE-PEG2000, DOPE, and CHEMS were dissolved in chloroform solutions and mixed at 0.5:6:4 molar ratio, respectively [41]. The organic solvent was removed under vacuum and nitrogen to afford a dry lipid film, which was hydrated under vigorous stirring with a solution of PEI-elastase /siRNA dissolved in PBS (3 mg/ml). Then, the suspension kept at 4℃ and extruded 20 times through a polycarbonate membrane filter of 500 nm, and 20 times through a polycarbonate membrane filter of 200 nm using an Avanti Hand Extruder (Avanti Polar Lipids). The P-E/S Lip were obtained through centrifugation (12,000 rpm, 10 min).

### Characterization of P-E/S lip

The hydrodynamic size and zeta potential of P-E/S Lip were measured by DLS using Zetasizer (Malvern Panalytical, UK). For morphology, P-E/S Lip were examined using CryoTEM in a FEI Talos F200C (Thermo Fisher Scientific In, USA).

### Reverse transcription qPCR analysis

RT-qPCR was performed to evaluate CD274 gene silencing in B16-F10 cells according to manufacturer’s instructions. The qPCR primers used in this experiment are given as follows:

PD-L1siRNA: 5’-GACUCAAGAUGGAACCUGA-3’

PD-L1_F_: 5’-TGCTGCATAATCAGCTACGG-3’

PD-L1_R_: 5’-CCACGGAAATTCTCTGGTTG-3’

GAPDH_F_: 5’- TGCACCACCAACTGCTTAGC − 3’

GAPDH_R_: 5’- GGCATGGACTGTGGTCATGAG − 3’

### Western blotting

B16-F10 and MCF-10 A cells were seeded into 6-well plates (2 × 10^5^ cells per well). After the cells had reached 80–90% confluency, the cells were treated with different liposomes. After incubating for 4 h, each group of cells was washed and lysed. The proteins were collected, quantified, and used next for western blotting according to the manufacturer’s instructions.

### Evaluation of cell apoptosis

Briefly, 2 × 10^6^ tumor cells were incubated with 10µM Calcein AM (CAM, Dojindo) in culture medium for 30 min at 37 °C protected from light. Then the culture medium containing CAM was removed and the cell pellets were washed five times with Dulbecco’s phosphate buffered saline without calcium or magnesium (DPBS). The tumor cell density was adjusted to 1 × 10^5^/ml with culture medium. 100 µl medium containing 1 × 10^4^ tumor cells were added into the 96-well plates. after different treatment for 6 hours, 50 µl of the culture supernatant was used to measure fluorescence intensity at 485 nm excitation and 530 nm emission wavelengths. Cytotoxicity was calculated according to the following formula: %Cytotoxicity = (Experimental-Target spontaneous)/ (Target maximum-Target spontaneous) ×100% [42].

### ICD evaluation of tumor cells induced by P-E/S lip

B16-F10 were seeded in 24-well dishes (2 × 10^5^ cells per well) and incubated overnight to reach 60 ~ 70% confluency. After different treatment for 48 h, the cell culture supernatant was collected and evaluated using an HMGB1 Assay Kit (Solarbio). For calreticulin staining, after 24 h treatment, the cells were washed with PBS and fixed with 4% paraformaldehyde, and staining with calreticulin according with the routine immunohistochemical staining process.

### Animal model

B16-F10 or 4T1 xenograft animal model was established by subcutaneously inoculating 5 × 10^5^ cells resuspended in PBS buffer (100 µl) into female C57BL/6J mice at the age of 6 weeks old. For further exploring the abscopal therapeutic effect on distal tumors, a bilateral tumor model was built. Briefly, 5 × 10^5^ cells were subcutaneously injected into the right hind legs of 6-week-old C57BL/6J, 7 days later, 2 × 10^5^ cells were injected into the left hind legs (2 tumor). To create lung colonization, B16-F10 cells (0.1 × 10^6^) were injected into the lateral tail vein of C57BL/6 mice. Lungs were excised and the number of tumors were quantified 14 days post injection.

### Tumor imaging in vivo

For tumor growth evaluation, mice were imaged using an IVIS Spectrum In vivo Imaging System. As mentioned above, 5 × 10^5^ B16-F10/Luc cells were inoculated to each mouse, following different treatments, mice were injected with 150 mg/kg D-luciferin intraperitoneally. Mice were placed in an imaging chamber in the supine position, and under 1–2% isoflurane gas anesthesia. Bioluminescence was measured and superimposed on photographic images of the mice. Images were analyzed using Living Image 4.3.1. The signal was calculated using fixed-volume regions of interest to estimate the tumor burden, and total flux was calculated.

### Biodistribution of P-E/S lip in vivo

When the tumor volume reached 200–500 mm^3^, the mice were used to investigate the tumor targetability. B16-F10 tumor-bearing mice were injected with Cy5-labeled liposome through the tail vein. At 24 h, in vivo fluorescence microscopy was performed using an IVIS Lumina II in vivo imaging system. After in vivo imaging, the mice were euthanized, and the major organs (tumor, liver, heart, lung, spleen, kidneys, and intestines) were dissected and imaged.

### Anti-tumor efficacy*in vivo*

When the tumor volume reached 50 ~ 100 mm^3^, mice were randomly divided into four groups (*n* = 4–8) and treated as follows: (i) PBS, (ii)P/S Lip, (iii)E/S Lip, (iv)P-E/S Lip. The injection volume of each solution was 100 µl at enzyme concentration of 0.6 mg/kg via intratumoral administration. The injection volume of each solution was 100 µl at enzyme concentration of 5 mg/kg via intravenous administration. The first day of intratumoral injection was considered as day 1. Tumor volumes and body weights were monitored every 2 days after the first injection. The tumor volume was calculated according to the formula V = 0.5ab^2^, where a and b are the long and short axes of the tumor (in millimeters), respectively. After treatment, mice were euthanized and the tumor tissues were imaged and weighed. Tumor tissues and major organs were also harvested for flow cytometry and immunohistochemistry assays.

### Statistical analysis

All data are shown as mean ± s.d. Each experiment was repeated independently at least three times unless otherwise indicated. Comparisons of two groups were performed by using unpaired two-tailed Students’ *t*-tests. For multiple comparisons, one-way analysis of variance (ANOVA) with a Tukey test was used when more than two groups were analysed. A *P* value < 0.05 was considered significant (*P < 0.05, **P < 0.01, ***P < 0.001).

### Electronic supplementary material

Below is the link to the electronic supplementary material.

Supplementary Material 1

## Data Availability

All data obtained throughout this study are presented in the manuscript or supporting information. All relevant data are available from the corresponding author upon reasonable request.
