# Supplementary material for: PD-L1 siRNA hitched polyethyleneimine-elastase constituting nanovesicle induces tumor immunogenicity and PD-L1 silencing for synergistic antitumor immunotherapy
Source: J Nanobiotechnology. 2024 Jul 27;22:442. doi: 10.1186/s12951-024-02700-4 (PMC11282766; doi:10.1186/s12951-024-02700-4)
Supplement: Supplementary file 1 — Supplementary Material 1 [file 12951_2024_2700_MOESM1_ESM.docx]

Supporting Information

PD-L1 siRNA hitched polyethyleneimine-elastase constituting nanovesicle induces tumor immunogenicity and PD-L1 silencing for synergistic antitumor immunotherapy

Li Du^1,2†^, Yao Gong^1†^, Xiaoying Zhang^1^, Jide Sun^3^, Fengxia Gao^1^, Meiying Shen^4^, Huili Bai^1^, Tiantian Yang^1^, Xiaoxue Cheng^1^, Siqiao Li^5^, Jian Peng^2^, Zhangling Liu^2^, Shijia Ding^6^, Junman Chen^6^* and Wei Cheng^1,2^*


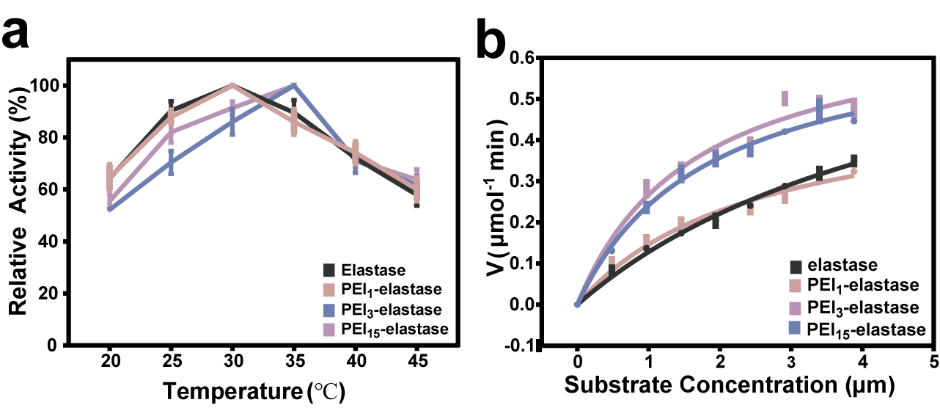


**Fig. S1** Kinetic characterization of elastase and PEI-elastase. (**a**) Enzymatic activities of elastase, PEI_1_-elastase, PEI_3_-elastase and PEI_15_-elastase at different temperatures. The optimum temperature was determined by the enzyme activity at the corresponding temperature, and the highest activity was plotted as 100%. (**b**) Michaelis-Menten curve for elastase, PEI_1_-elastase, PEI_3_-elastase and PEI_15_-elastase, with casein concentrations plotted on the x axis and velocity plotted on the y axis.


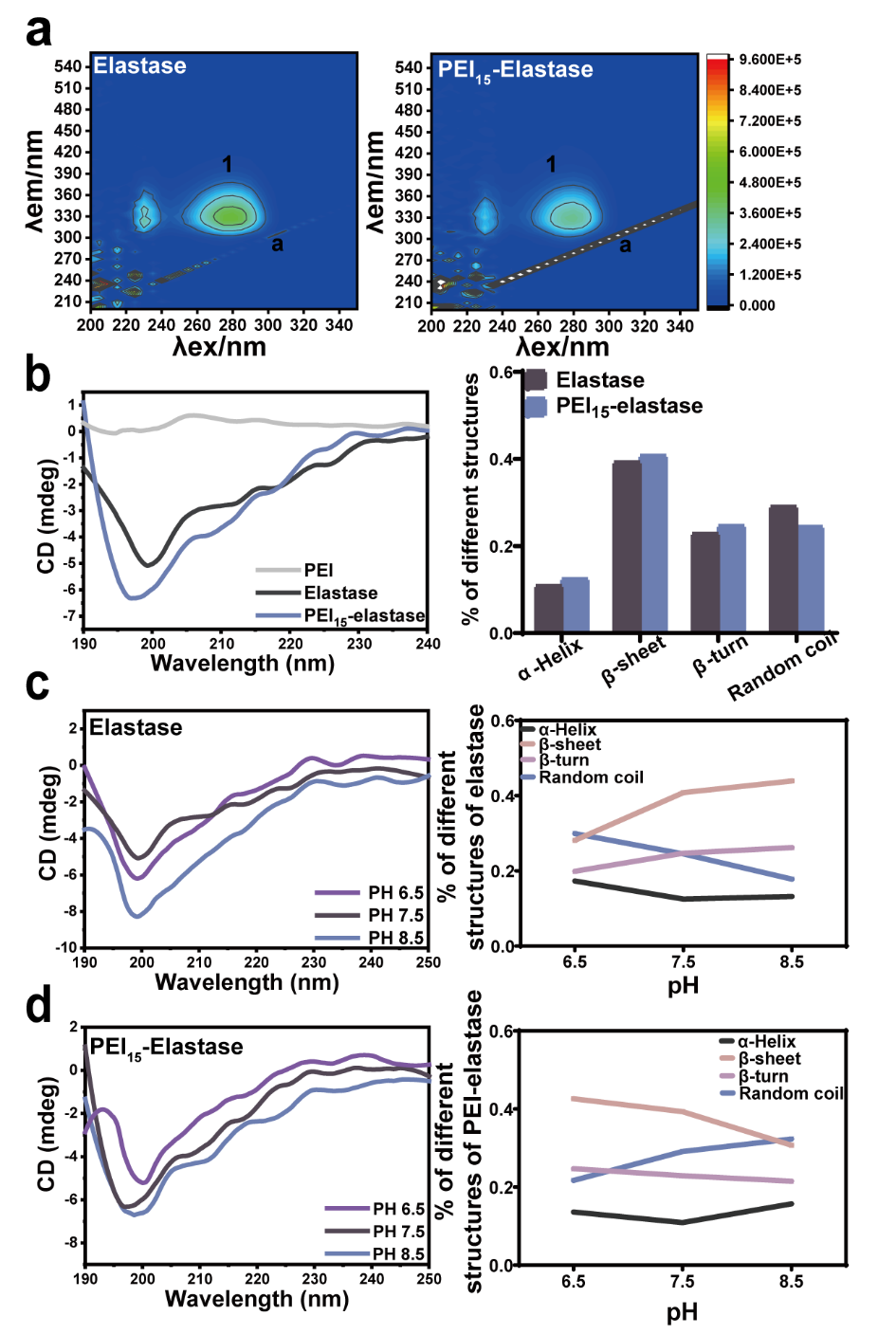


Fig. S2 characterization of elastase and PEI_15_-elastase. (a) Three-dimensional fluorescence spectra of elastase and PEI_15_-elastase. (b) The Circular dichroism (CD) spectroscopy spectra of elastase and PEI_15_-elastase. The percentage of secondary structural elements of elastase and PEI-elastase, obtained by deconvoluting the CD spectra using CDNN software. (c-d) CD spectra of elastase and PEI_15_-elastase at different pH in the scan range from 190 to 250 nm. The percentage of secondary structural elements of elastase and PEI-elastase at varying pH, obtained by deconvoluting the CD spectra using CDNN software.


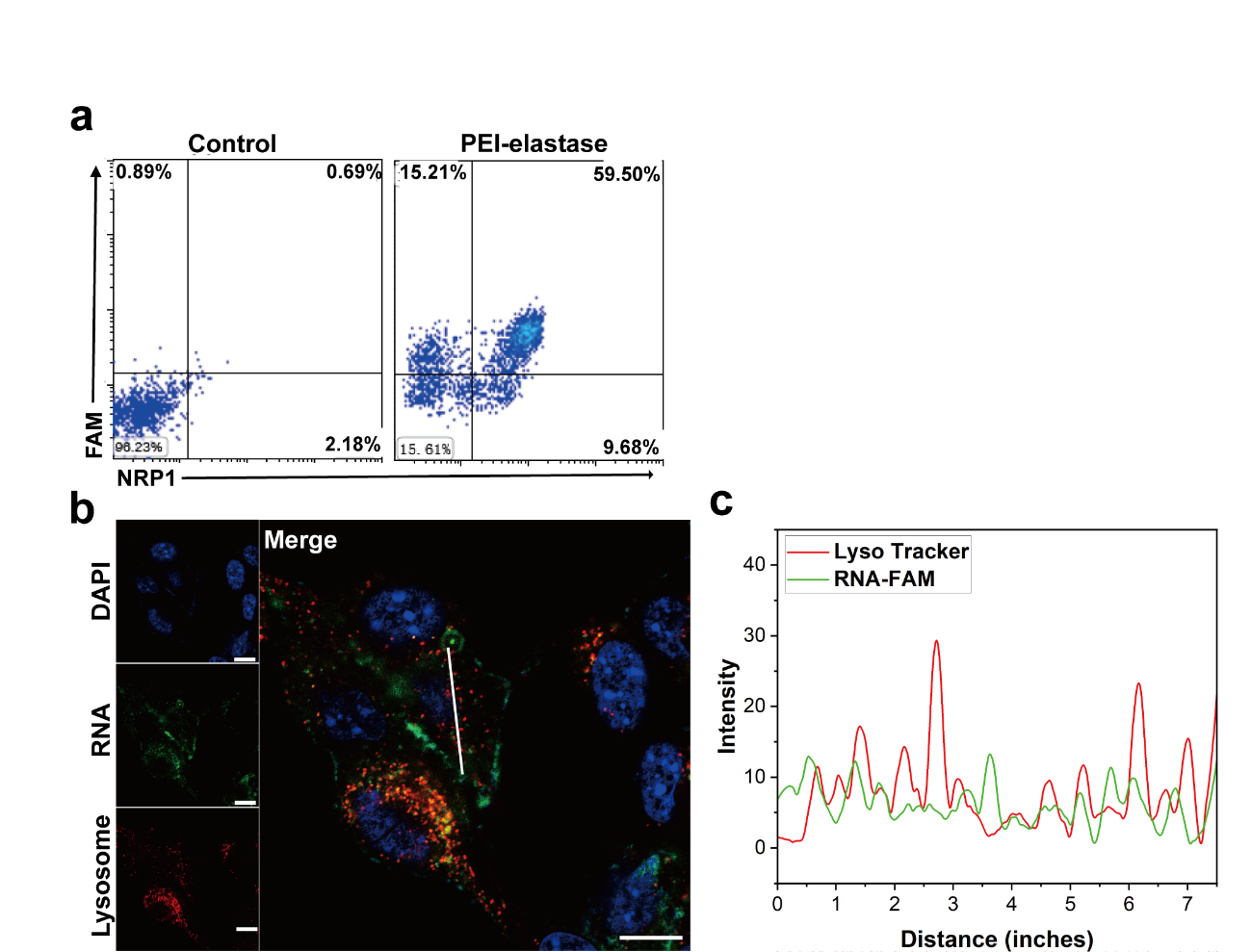


Fig. S3 PEI-elastase-mediated gene delivery. (a) Flow cytometry analysis of NRP1 and FAM expression on MDA-MB-231 cells after incubated with PEI-elastase/FAM-DNA for 2 hours. (b, c) CLSM images of MDA-MB-231 cells treated with PEI-elastase/RNA-FAM for 4 hours. The nuclei and acidic compartments (endosomes and lysosomes) were stained with Hoechst (blue) and Lyso Tracker (red), respectively. Scale bars: 5 μm.


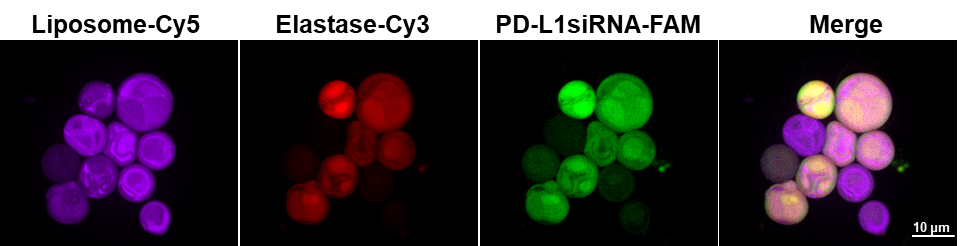


Fig. S4 Co-localization validation of P-E/S Lip. The liposomes were labeled with Cy5; elastase was labeled with Cy3 and PD-L1siRNA was labeled with FAM. CLSM images showing co-localization of the three fluorescence colors suggested that the PEI-Elastase/PD-L1siRNA was successfully encapsulated with liposome membrane (before liposome extrusion).


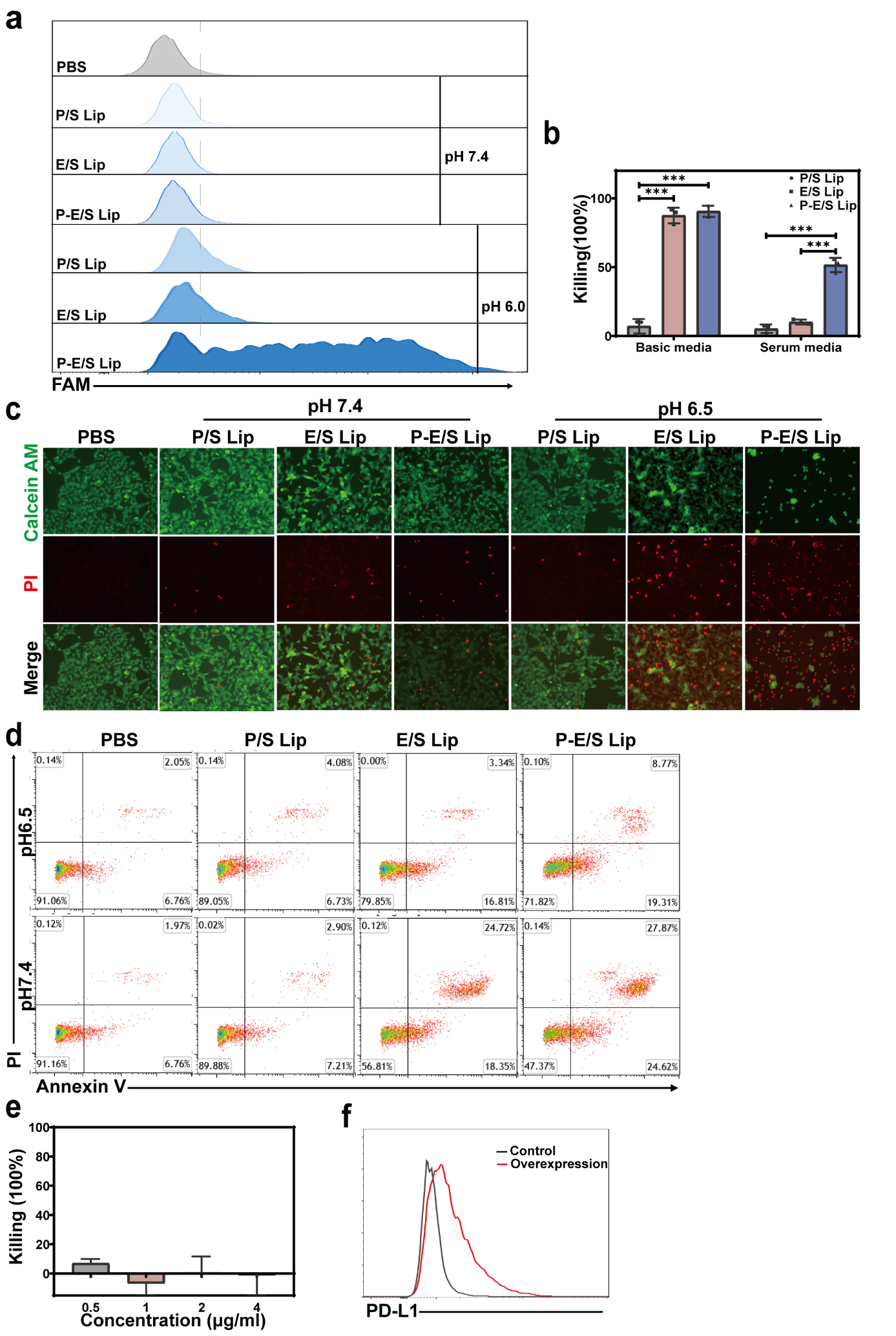


Fig. S5 Synthesis and cytotoxicity of P-E/S Lip. (a) Flow cytometry analysis of the internalization of FAM-labeled PD-L1siRNA (green) after incubation with different liposomes for 2 hours. (b) Cytotoxicity of different liposomes on MDA-MB-231 cells measured by Calcein AM release assay. All data are presented as mean ± *s.d.* (c) images of PI and Calcein AM of the B16-F10 cells after incubated with different liposomes at pH 7.4 or pH 6.5 for 6 hours, respectively. (d) Apoptosis rate of B16-F10 cells determined by flow cytometry using Annexin V-FITC/PI apoptosis assay following different treatments for 6 hours. (e) Cytotoxic activity of P-E/S Lip against human lymphocytes at various concentrations assessed by Calcein AM release assay. (f) Flow cytometry analysis of PD-L1 level in B16-F10 cells after PD-L1 overexpression. Data are presented as mean ± s.d. from three independent experiments (n = 3). *P* values were determined by one-way ANOVA with Tukey test (**b**). ****p* < 0.001.


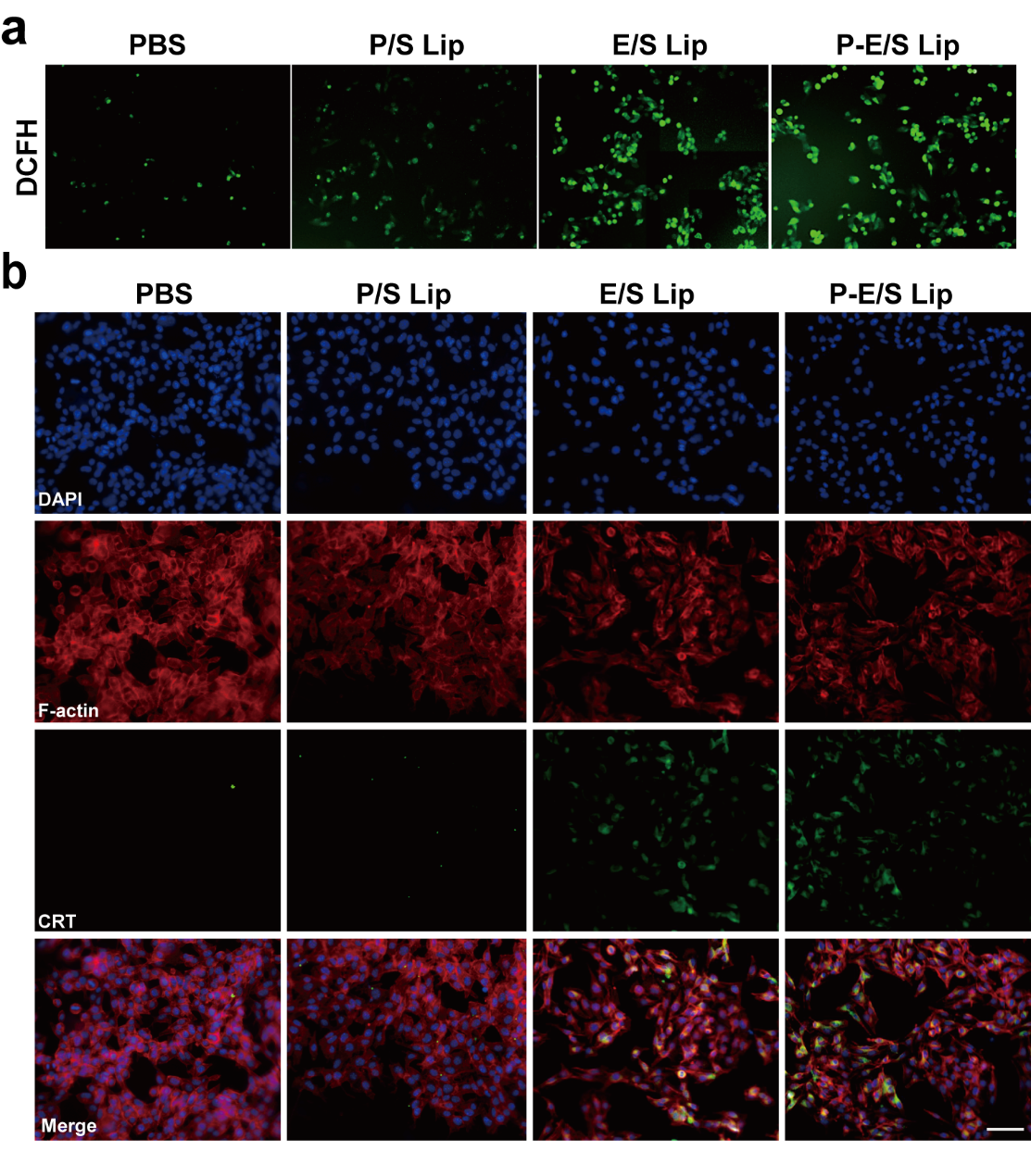


Fig. S6 ICD effect induced by P-E/S Lip. (a) Images of ROS expression of B16-F10 cells after treating with different liposomes at pH 6.5 for 3 hours. The production of ROS in the tumor was measured with Dichlorodihydrofluorescein Diacetate (DCFH) using fluorescence microscope. (b) CLSM images of CRT expression on B16-F10 cells after treating with different liposomes at pH 6.5 for 24 hours. Cell cytoskeleton F-actin and cell nuclei were counterstained with rhodamine phalloidin (red) and 4,6-diamino-2-phenyl indole (DAPI) (blue), respectively. The scale bars: 50 μm.


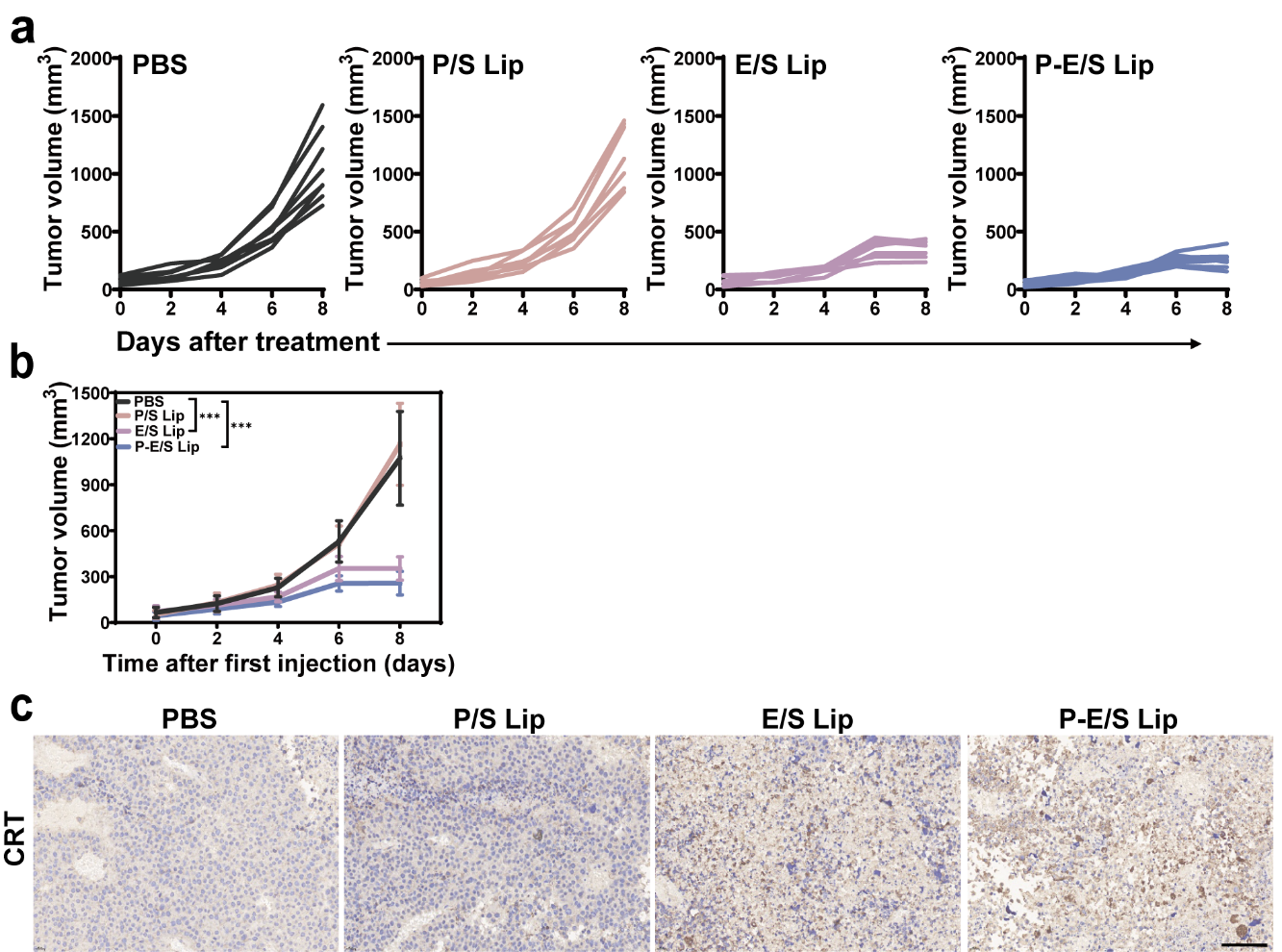


Fig. S7 Antitumor activities of P-E/S Lip via intratumoral administration. (a-b) Curves of tumor growth in B16-F10 tumor-bearing mice after different treatments (n = 5-8). (c) Immunohistochemical evaluation of the CRT expression in primary tumor sections following various treatments, Scale bar: 50 μm. Data are presented as mean ± s.d. *P* values were determined by one-way ANOVA with Tukey test (**b**). ****p* < 0.001.


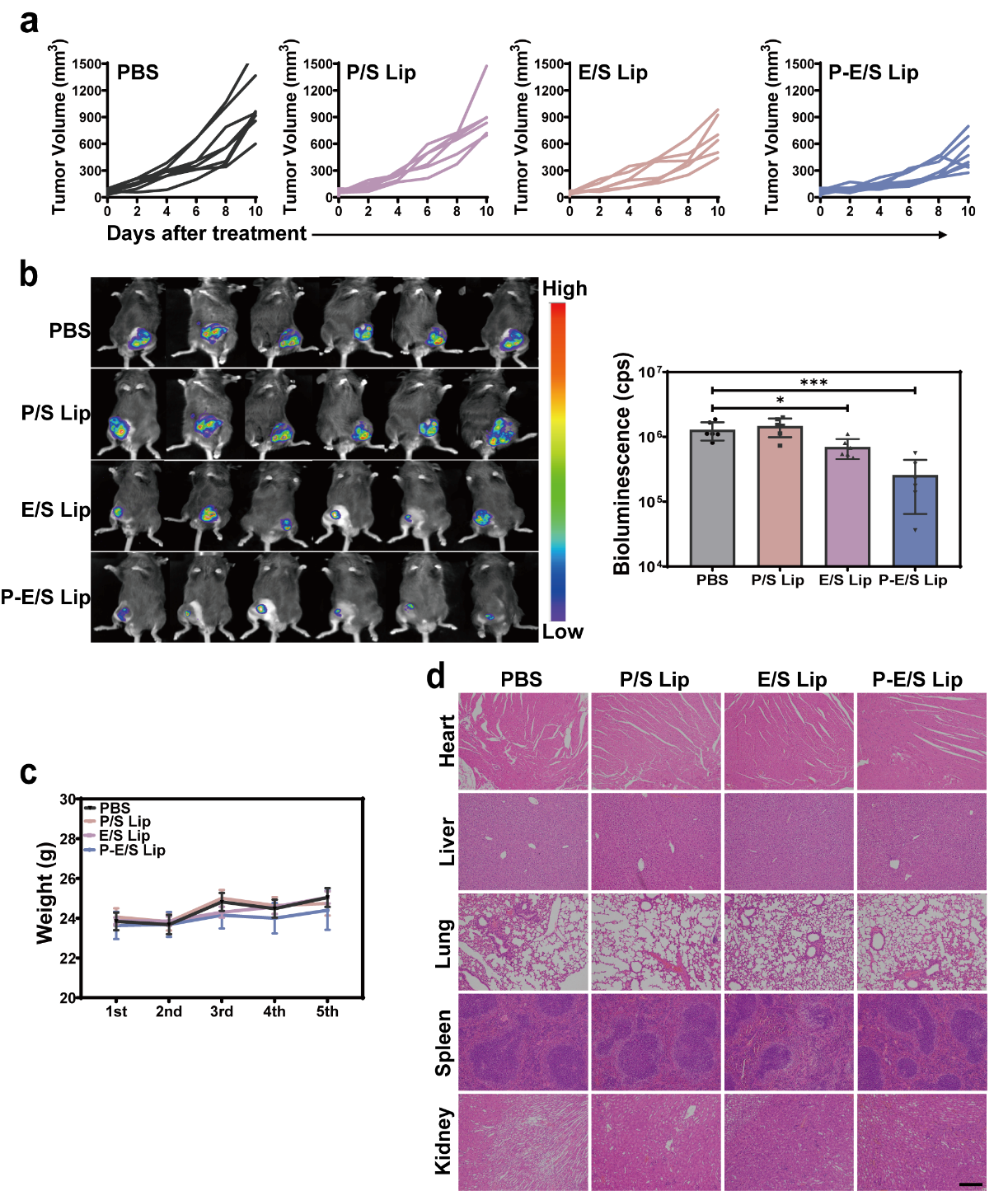


Fig. S8 Antitumor efficacy of P-E/S Lip via intravenous administration. (a) Curves of tumor growth in B16-luc tumor-bearing mice after systemic administration of different liposomes. (b) In vivo bioluminescence imaging of orthotopic B16-luc tumor-bearing mice with different treatments at 14 days (n = 6). Quantitative bioluminescence (radiance = photons/sec/cm2/sr) imaging data for all mice are shown. (c) Body weight change of mice treated with different liposomes. (d) HE staining pictures of visceral tissue of B16-F10 tumor-bearing mice with different treatments at 14 days, the scale bars: 200μm. Data are presented as mean ± s.d. *P* values were determined by one-way ANOVA with Tukey test (**b**). **p* < 0.05, ****p* < 0.001.


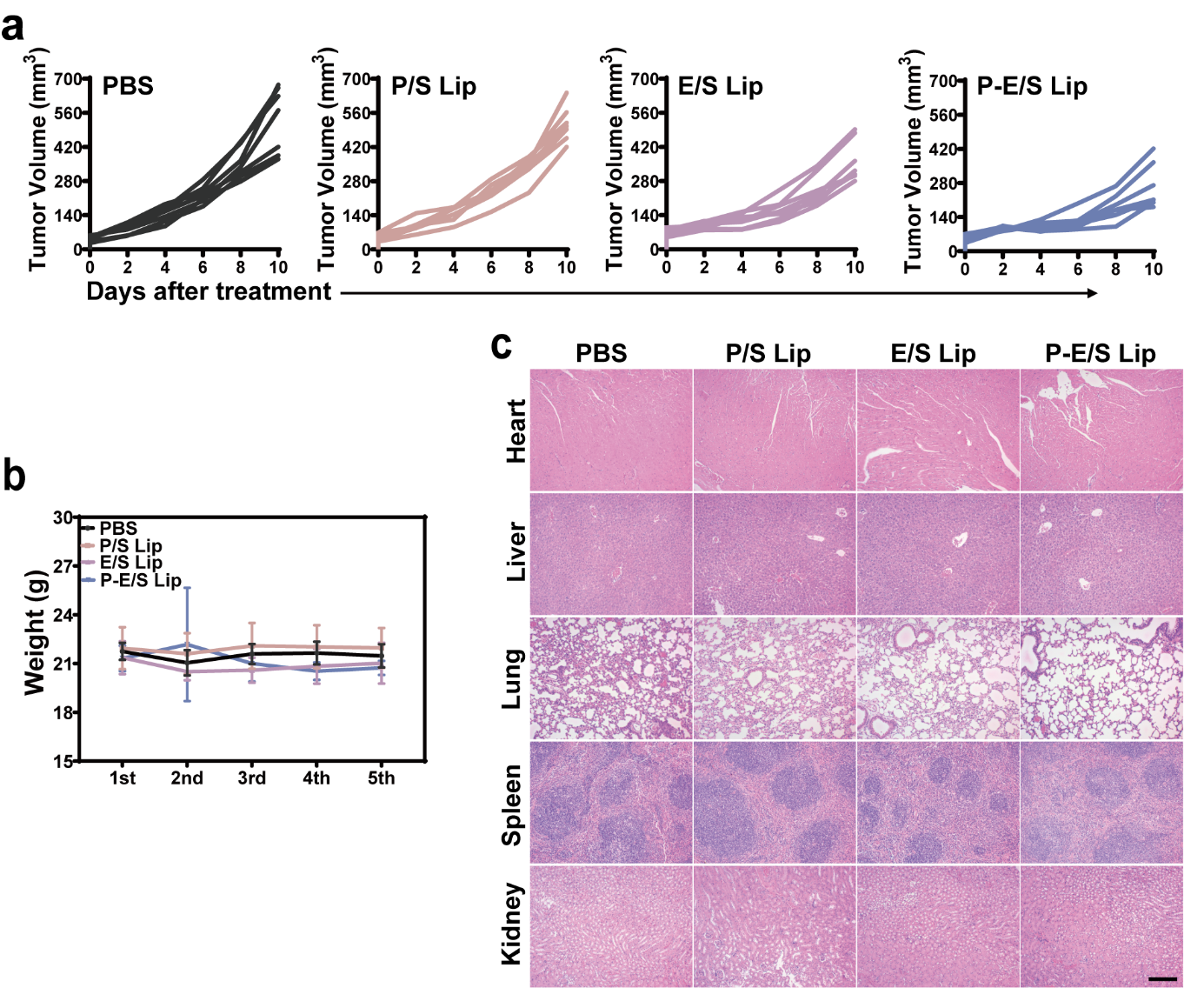


Fig. S9 Evaluation of the 4T1 tumor inhibition effect of P-E/S Lip. (a) Curves of tumor growth in 4T1 tumor-bearing mice after systemic administration of different liposomes. (b) Body weight change of mice treated with different liposomes. (c) HE staining pictures of visceral tissue of 4T1 tumor-bearing mice with different treatments at 14 days, the scale bars: 200μm.
